# Estimation of model accuracy in CASP15 using the ModFOLDdock server

**DOI:** 10.1002/prot.26532

**Published:** 2023-06-14

**Authors:** Nicholas S. Edmunds, Shuaa M. A. Alharbi, Ahmet G. Genc, Recep Adiyaman, Liam J. McGuffin

**Affiliations:** ^1^ School of Biological Sciences University of Reading Reading UK

**Keywords:** 3D modeling, EMA, estimates of model accuracy, model quality assessment, protein structure prediction, QA, quaternary structure

## Abstract

In CASP15, there was a greater emphasis on multimeric modeling than in previous experiments, with assembly structures nearly doubling in number (41 up from 22) since the previous round. CASP15 also included a new estimation of model accuracy (EMA) category in recognition of the importance of objective quality assessment (QA) for quaternary structure models. ModFOLDdock is a multimeric model QA server developed by the McGuffin group at the University of Reading, which brings together a range of single‐model, clustering, and deep learning methods to form a consensus of approaches. For CASP15, three variants of ModFOLDdock were developed to optimize for the different facets of the quality estimation problem. The standard ModFOLDdock variant produced predicted scores optimized for positive linear correlations with the observed scores. The ModFOLDdockR variant produced predicted scores optimized for ranking, that is, the top‐ranked models have the highest accuracy. In addition, the ModFOLDdockS variant used a quasi‐single model approach to score each model on an individual basis. The scores from all three variants achieved strongly positive Pearson correlation coefficients with the CASP observed scores (oligo‐lDDT) in excess of 0.70, which were maintained across both homomeric and heteromeric model populations. In addition, at least one of the ModFOLDdock variants was consistently ranked in the top two methods across all three EMA categories. Specifically, for overall global fold prediction accuracy, ModFOLDdock placed second and ModFOLDdockR placed third; for overall interface quality prediction accuracy, ModFOLDdockR, ModFOLDdock, and ModFOLDdockS were placed above all other predictor methods, and ModFOLDdockR and ModFOLDdockS were placed second and third respectively for individual residue confidence scores. The ModFOLDdock server is available at: https://www.reading.ac.uk/bioinf/ModFOLDdock/. ModFOLDdock is also available as part of the MultiFOLD docker package: https://hub.docker.com/r/mcguffin/multifold.

## 1 INTRODUCTION

The critical assessment of structure prediction (CASP) competitions have been running on a biannual basis since 1994, with the latest meeting (CASP15) held in the summer of 2022. The CASP organizers have included a blind multimeric assembly modeling category in their experiments since 2016 and have formed close collaborations with the CAPRI (critical assessment of predicted interactions) organizers.

CASP15 differed from previous competitions in several ways; a much greater emphasis was put on assembly targets (41 compared to 22 in CASP14) with 37 of these being shared CAPRI targets. CASP15 also represented the first competition since AlphaFold^1^ led the CASP14 tertiary structure prediction category by some distance, achieving an unprecedented, summed *Z*‐score of 244 compared to the next best group with 92.1.^2^ As such, there was much anticipation as to whether a similarly dramatic increase in multimeric quality would occur at CASP15.

In fact, although there was a 31% increase in assembly accuracy and a 7% increase in native‐like predictions (https://predictioncenter.org/casp15/), the naïve AlphaFold2‐Multimer complex models (NBISAF2‐Multimer, group 390), which were submitted as a baseline, were ranked 31st overall (*Z*‐score of 12.3) with the winning group scoring 35.4 and the top‐ranked server group (in 4th place overall) scoring 25.0 (https://predictioncenter.org/casp15/zscores_multimer.cgi). It is clear from these results that the improvements seen in tertiary structure modeling quality have not yet fully translated to assembly or quaternary structure and no single approach has come close to solving the protein complex modeling challenge.

For this reason, objective, and accurate model quality assessment (QA) remains a crucial component in the drive to improve the quality of protein complex models. Perhaps in recognition of this, CASP15 was the first meeting to include a specific model QA category focusing solely on quaternary structure models, superseding the estimation of model accuracy (EMA) categories in previous CASPs, which were only for predicted tertiary structures. The new EMA category was focused solely on the scoring of multimeric assemblies and required submissions within 48 h of the release of each model population. Competing groups were required to submit scores for their chosen best model in either the QMODE1 or QMODE2 format. Both formats required an overall global score (SCORE) to reflect the overall accuracy estimate of the model and there was also a second, optional, score (QSCORE) to reflect the overall accuracy of the interface. The QMODE2 format additionally required a series of individual residue‐level confidence scores to reflect the likelihood of their inclusion in the interface.

In this paper, we report on the performance of the ModFOLDdock server, which has been developed to offer a quaternary structure equivalent to our popular ModFOLD server.^3^ In a similar way to the latest ModFOLD versions, the ModFOLDdock server adopts a consensus approach incorporating a range of single‐model and clustering based algorithms. The ModFOLDdock server was developed for CASP15 with three separate variants, each optimized for different facets of the quality estimation problem. First, ModFOLDdock was optimized for positive linear correlations with observed scores. Second, ModFOLDdockR was optimized for ranking, that is, top1‐ranked models have higher observed scores but the relationship between predicted and observed scores may not be linear. Lastly, ModFOLDdockS was designed as a quasi‐single model method, where submitted models were scored individually against a set of reference models generated using our MultiFOLD^4^ server.

### 1.1 How does ModFOLDdock differ from other state‐of‐the‐art methods?

Traditionally, there have been two principal categories of model QA programs for tertiary structures; those that score individual models (single‐model methods) and those which compare multiple models to form consensus scores (clustering methods). Based on a review of the CASP15 abstracts, the other top‐quality estimation methods for quaternary structure models that were tested at CASP15 include: MULTICOM_qa^5^—a consensus method of pairwise MMalign^6^ comparisons; VoroIF^7^—a single model method including a graph attention network that accepts a Voronoi tessellation‐derived graph of interface contacts; and GuijunLab‐RocketX^8^—a single model method using the latest version of DeepUMQA^9^—an Ultrafast Shape Recognition‐based system using deep learning.

The ModFOLDdock server methods aim to increase prediction accuracy by combining the output from a number of individual consensus and single‐model scoring algorithms. Our approach, therefore, overlaps with aspects of many of the above‐listed methods by calculating a consensus of contributing scores, including established single model methods (voronota‐js‐voromqa^10^), deep‐learning based methods (e.g., the CDA‐score^11^, ^12^), as well as several clustering‐based measures.

## 2 MATERIALS AND METHODS

### 2.1 Development of ModFOLDdock

ModFOLDdock has been developed through the repeated improvement of an original legacy version which calculated an unweighted mean (consensus) score based on four distance‐based scoring methods. The latest version, developed for CASP15, underwent major modifications in terms of both the contributing component predicted scores and the optimization to the target observed scores.

### 2.2 The component scores

Our new ModFOLDdock server uses various combinations of seven individual scoring methods: ModFOLDIA, our own clustering interface accuracy score; DockQJury, a clustering approach based on the DockQ^13^ score; QSscoreJury and QSscoreOfficialJury, clustering approaches using QS‐scores;^14^, ^15^ lDDTOfficialJury, clustering using lDDT^16^ scores; voronota‐js‐voromqa, the Voronoi tessellation score^10^ and the CDA‐score, our contact distance agreement score.

The decision to include voronota‐js‐voromqa and a version of our CDA‐score adapted for multimers was, in part, informed by the success in CASP and CAMEO competitions of our tertiary structure QA method ModFOLD, which includes variants of both of these scores among its inputs. It was also deemed important to integrate single‐model methods as they are superior to clustering methods in cases when there are few variations between models or when only few models are considered. The output scores from all methods ranged between 0 and 1, with higher scores implying higher quality. Further detail on our ModFOLDIA and CDA scores as well as how the other scores were calculated, can be found in Supplementary Materials.

### 2.3 The target observed scores

The CASP assessors routinely rank competing groups on the basis of their combined calculated *Z*‐scores. The CASP14 sum of *Z*‐scores for overall group rankings for multimers was calculated on an unweighted basis as; *Z*‐score(F1) + *Z*‐score(Jaccard) + *Z‐*score(Oligo‐lDDT) + *Z*‐score(TM).^17^

A *Z*‐score can be considered a statistically normalized version of the raw score, and it follows that the higher the raw score value, the higher the *Z*‐score. On this basis, we reasoned that we could use the raw CASP observed scores for multimers to calculate two target scores as benchmarks against which the ModFOLDdock predicted scores could be optimized. Thus, the target observed scores were calculated using CASP assessors' formulae as follows:

Interface: an unweighted mean of ICS (F1) and IPS (Jaccard, Coeff.).

Fold: an unweighted mean of Oligo‐lDDT and TM‐score.

### 2.4 The CASP15 QA QMODE2 format

**FIGURE 1:**
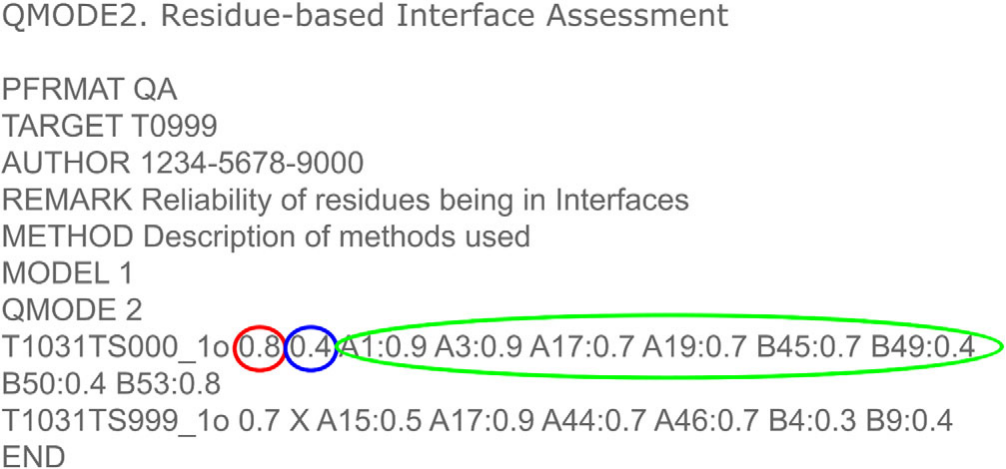
The CASP15 QMODE2 scoring requirement for the estimation of model accuracy category. Ringed in red: the overall global fold score (SCORE); ringed in blue: the overall interface score (QSCORE) and ringed in green: the individual residue confidence scores.

As stated in the introduction, the EMA category of CASP15 required the production of three separate scores. Figure 1 shows the required format for the QA QMODE2 submissions with the positioning of each score including the overall global model quality score (SCORE), the overall quality of the interface score (QSCORE) and the set of individual interface confidence scores, which estimate the likelihood of the named residue contributing to the interface (https://predictioncenter.org/casp15/index.cgi?page=format#QA).

### 2.5 The ModFOLDdock variants

**FIGURE 2:**
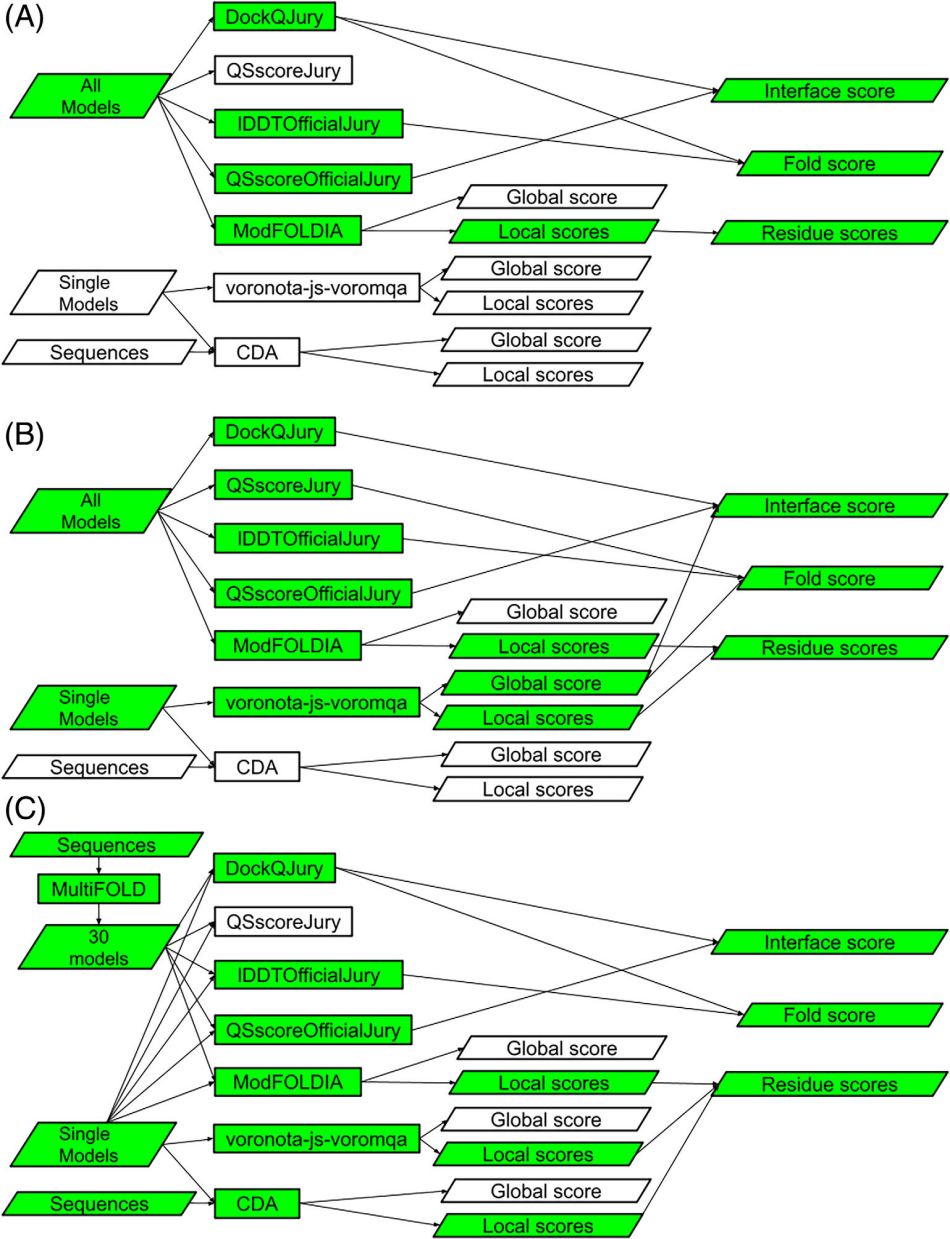
Flowcharts showing the constituent component methods and their contributions to the consensus scores for the three ModFOLDdock variants. (A) ModFOLDdock. (B) ModFOLDdockR. (C) ModFOLDdockS. Green colored boxes indicate the scores that contribute directly to the overall global fold (SCORE), overall interface (QSCORE) and individual residue confidence scores.

Three variants of ModFOLDdock were ultimately developed and these were used as QA methods within our MultiFOLD^4^ modeling pipeline as well as the CASP15 EMA category. The flowcharts for each of the ModFOLDdock variants show how the inputs, methods and outputs are all connected to produce the various quality scores for each model (Figure 2). Table 1 shows the methods contributing to each QMODE2 score for each ModFOLDdock variant.

**TABLE 1:** The component scores that contribute to each score in the CASP15 QMODE2 files for each ModFOLDdock variant.

Variant	Fold	Interface	Residue
ModFOLDdock	DockQJury, lDDTOfficialJury	DockQJury, QSscoreOfficialJury	ModFOLDIA
ModFOLDdockR	QSscoreJury, lDDTOfficialJury, voronota‐js‐voromqa	DockQJury, QSscoreOfficialJury, voronota‐js‐voromqa	voronota‐js‐voromqa, ModFOLDIA
ModFOLDdockS	DockQJury, lDDTOfficialJury	DockQJury, QSscoreOfficialJury	CDA, voronota‐js‐voromqa, ModFOLDIA

Prior to CASP15, we optimized the combinations of scores used in each ModFOLDdock variant using the CASP14 data set of multimer models (see details for each variant below). Figure S1 shows how the performance of each individual scoring method compares with the combined scores used by each variant in terms of correlations and ranking based on both the interface and fold accuracy scores. Tables S1 and S2 show the data for every combination of the seven component scores in terms of correlation and ranking performance, respectively.

#### 2.5.1 ModFOLDdock

For the ModFOLDdock variant, the predicted global scores were optimized for positive linear correlations with observed scores according to assessors' formulae for CASP14 multimer models.^17^ The overall fold accuracy score (SCORE) was calculated from the mean of DockQJury and lDDTOfficialJury component scores. The overall interface accuracy (QSCORE) was calculated from the mean of DockQJury and QSscoreOfficialJury scores. Individual residue confidence scores were calculated using the ModFOLDIA method (Figure 2A).

#### 2.5.2 ModFOLDdockR

For ModFOLDdockR, the predicted global scores were optimized for ranking, meaning the top1‐ranked models should have higher observed scores. As such, the relationship between predicted and observed scores may not be linearly correlated. The overall fold accuracy (SCORE) was calculated from the mean of QSscoreJury, lDDTOfficialJury, and voronota‐js‐voromqa scores. The overall interface accuracy score (QSCORE) was calculated from the mean of DockQJury, QSscoreOfficialJury, and voronota‐js‐voromqa scores. Individual residue confidence scores were calculated from the mean of ModFOLDIA and per‐residue voronota‐js‐voromqa scores (Figure 2B).

#### 2.5.3 ModFOLDdockS

The ModFOLDdockS variant used a quasi‐single model approach where 30 reference multimer models were generated from the input target sequence using our MultiFOLD modeling method (Figure S2) with each model then compared against the reference set using the seven individual scoring methods described above. The overall fold accuracy (SCORE) was calculated from the mean of DockQJury and lDDTOfficialJury scores, and the overall interface accuracy score (QSCORE) was calculated from the mean of DockQJury and QSscoreOfficialJury scores, that is, the same combinations used by ModFOLDdock. Individual residue confidence scores were calculated from the mean of ModFOLDIA, voronota‐js‐voromqa, and CDA scores (Figure 2C).

### 2.6 Processing large structures

For large structures (>1500 total residues), it was not always possible to carry out all‐against‐all pairwise comparisons within the 48 h window due to CPU and RAM limitations. In these cases, for ModFOLDdock and ModFOLDdockR, we initially scored all models using voronota‐js‐voromqa and thereafter selected the top 40 models to act as the reference set for the subsequent model comparisons.

## 3 RESULTS

### 3.1 Correlations of ModFOLDdock scores with CASP15 observed scores

**FIGURE 3:**
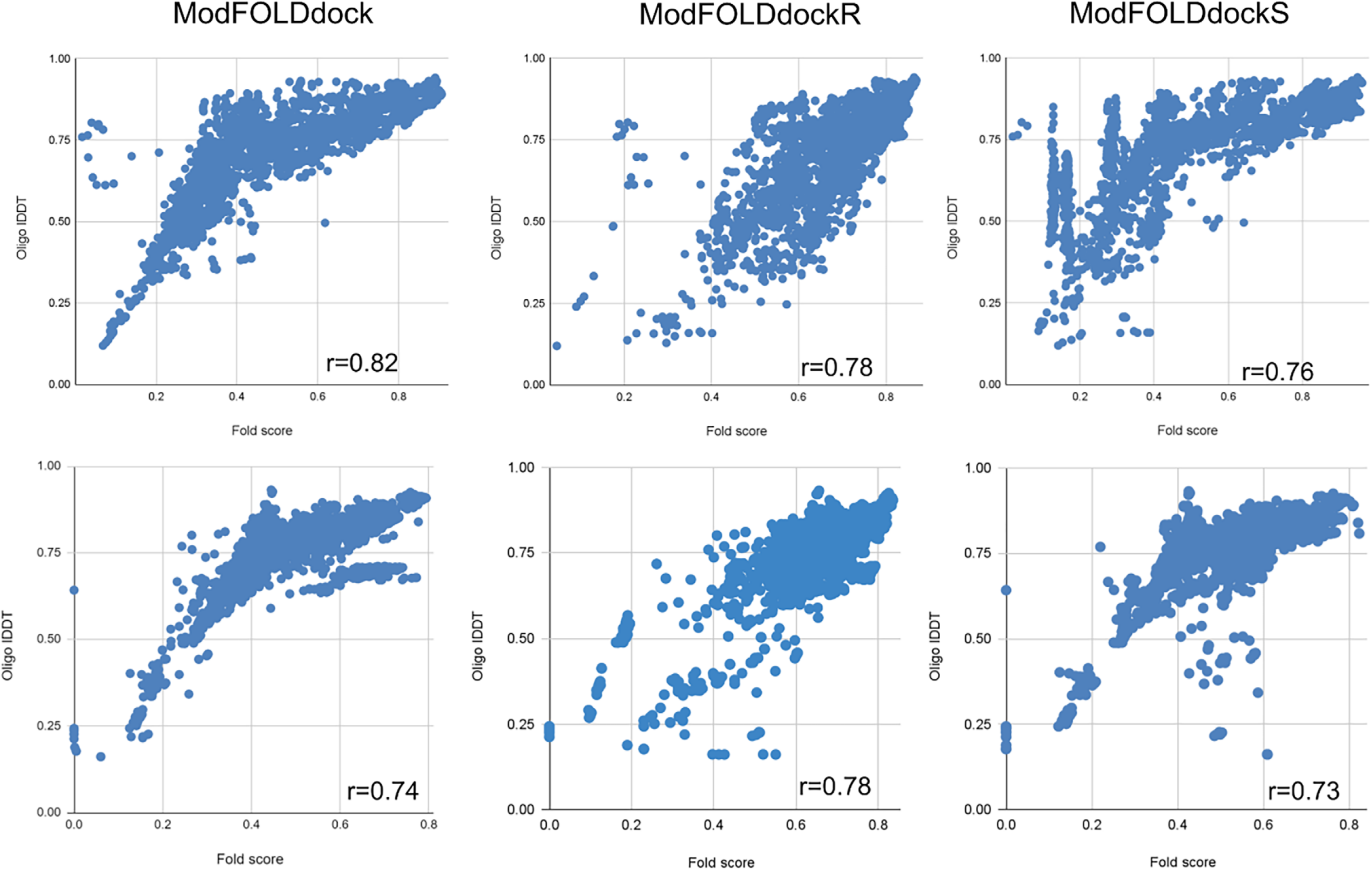
Scatter plots for ModFOLDdock variants predicted global fold scores (*x*‐axis) versus CASP15 oligo‐lDDT scores (*y*‐axis). Upper row: plots and Pearson *R* figures for all *homomeric* targets for variants ModFOLDdock (left), ModFOLDdockR (center) and ModFOLDdockS (right). Lower row: plots and Pearson R figures in the same left to right variant order for all *heteromeric* targets.

Figure 3 shows scatter plots for the overall global fold score for all three ModFOLDdock variants versus the observed oligo‐lDDT scores for the CASP15 models. The three plots forming the upper row show strong positive Pearson correlations achieved between the predicted and observed model quality scores for all homomeric targets, excluding T1160 and T1161. When the models for T1160 and T1161 are included in the plots, they show up as an obvious set of outliers and greatly affect the correlation scores (Figure S3). Targets T1160 and T1161 have similar sequences but different conformations due to crystallization conditions and the ModFOLDdock methods are unable to discriminate between models for these targets, with the observed scores being much lower than predicted.

Similarly, the lower row of Figure 3 shows strong positive correlations achieved between the predicted and observed model quality scores for all heteromeric targets, excluding H1171 and H1172. Again, these targets each have at least two alternative conformations, which greatly affect the correlation scores (Figure S4). The Pearson *R* values are at or above 0.73 for each ModFOLDdock variant and this is maintained across homomeric and heteromeric model populations when the few targets with alternative conformations are excluded.

### 3.2 CASP15 EMA rankings

The high levels of agreement between the ModFOLDdock predicted and CASP observed scores for most targets (Figure 3) translated into successful performance in the EMA category of CASP15. At least two of the ModFOLDdock variants were consistently ranked within the top five methods with one variant consistently ranked in the top two methods (Table 2, Figure S5).

**TABLE 2:** Summary of rankings from the official CASP15 estimation of model accuracy evaluation of predicted versus observed model quality scores.

(A) Fold Group Name	GDT_TS‐like		TM		Assessor based formula
	Pearson	ROC AUC	Pearson	ROC AUC	
MULTICOM_qa	0.629	0.689	0.712	0.703	2.189
AssemblyConsensus	0.643	0.688	0.635	0.706	2.131
ModFOLDdock	0.613	0.677	0.636	0.684	2.102
ModFOLDdockR	0.565	0.679	0.635	0.679	2.037
Venclovas	0.53	0.676	0.49	0.68	1.963

(B) Interface Group Name	DockQ‐wave		QS		Assessor based formula
	Pearson	ROC AUC	Pearson	ROC AUC	
AssemblyConsensus	0.685	0.703	0.765	0.734	2.312
ModFOLDdockR	0.624	0.694	0.673	0.695	2.164
ModFOLDdock	0.628	0.691	0.673	0.692	2.142
ModFOLDdockS	0.549	0.668	0.603	0.688	1.851
VoroIF	0.535	0.66	0.563	0.668	1.798

(C) Local Group Name	PatchDockQ		lDDT		Assessor based formula
	Pearson	ROC AUC	Pearson	ROC AUC	
GuijunLab‐RocketX	0.432	0.698	0.564	0.755	4.722
ModFOLDdockR	0.45	0.652	0.476	0.681	4.317
ModFOLDdockS	0.388	0.635	0.455	0.674	4.151
VoroIF	0.415	0.675	0.333	0.664	4.047
Venclovas	0.415	0.675	0.332	0.664	4.044

*Note*: Only the top 5 groups are shown. Raw score data are from https://predictioncenter.org/casp15/qa_global_fold.cgi. The “Assessor based” formulae produce similar rankings to the official ones but using all raw scores: https://predictioncenter.org/casp15/zscores_EMA.cgi. (A) Rankings for Global fold (total 22 groups). Rows are sorted by the “Assessor based” formula calculated as: (0.5*Pearson(GDT_TS)) + (0.5*Spearman(GDT_TS)) + AUC(GDT_TS)‐Loss(GDT_TS) + (0.5*Pearson(TM)) + (0.5*Spearman(TM)) + AUC(TM)‐Loss(TM). (B) Rankings for global interface score (total 17 groups). Rows are sorted by the “Assessor based” formula calculated as: (0.5*Pearson(DockQ‐wave)) + (0.5*Spearman(DockQ‐wave)) + AUC(DockQ‐wave)‐Loss(DockQ‐wave) + (0.5*Pearson(QS)) + (0.5*Spearman(QS)) + AUC(QS)‐Loss(QS). (C) Rankings for local residue score (total 13 groups). Rows are sorted by the “Assessor based” formula calculated as: (0.5*Pearson(PatchDockQ)) + (0.5*Spearman(PatchDockQ)) + AUC(PatchDockQ) + (0.5*Pearson(PatchQS)) + (0.5*Spearman(PatchQS)) + AUC(PatchQS) + (0.5*Pearson(CAD)) + (0.5*Spearman(CAD)) + AUC(CAD) + (0.5*Pearson(lDDT)) + (0.5*Spearman(lDDT)) + AUC(lDDT).

Table 2 shows the top 5 ranked methods for the three CASP15 EMA score categories as described in the Section 2 on the QMODE2 format. Table 2A shows the rankings for the overall global fold score (SCORE), where the ModFOLDdock variant was placed in second place behind MULTICOM_qa, with the ModFOLDdockR variant in third place. Table 2B shows the rankings for the overall interface score (QSCORE) showing that no single group outperformed either of the three ModFOLDdock variants with only the CASP15 assessor's assembly consensus score being greater in value. Table 2C shows the rankings for local residue confidence scores, again showing two ModFOLDdock variants in second and third place, this time with only Guijun RocketX performing better. Methods from no other group are as highly ranked across all three score categories as those from the ModFOLDdock server.

## 4 DISCUSSION

Overall, the ModFOLDdock server variants were arguably the most successful model QA methods at CASP15, being consistently placed in the top few groups across all aspects of the EMA category of CASP15. By our own analysis, the ModFOLDdock variants were also found to highly correlate with the CASP official observed scores (e.g., oligo‐lDDT) ranging from 0.73 to 0.82 as measured by the Pearson correlation coefficients. Furthermore, strong correlations were maintained across both homomeric and heteromeric model populations.

This success was achieved through the optimization of a hybrid consensus approach. A range of individual quality scores was selected based on their added value to the unweighted consensus and then optimized in a two‐stage process versus the CASP14 assessors' formulae for multimer ranking. First, a combination of quality scores for the ModFOLDdock variant was selected that produced optimal correlations with the observed scores. Second a combination of quality scores was selected for the ModFOLDdockR variant, which optimized for ranking the best models at the top, that is, selecting the highest quality top models according to the observed scores. Furthermore, the limitations of clustering‐based approaches were recognized and therefore a third variant (ModFOLDdockS) was developed using a quasi‐single model approach.

As demonstrated by the format of the EMA category at CASP15, where multiple scores were required to score models, there remain some important challenges involved in predicting the quality of protein assemblies. In addition to the relative strengths and weaknesses of the overall fold accuracy scores (e.g., TM‐scores vs. superposition independent scores lDDT), there is also the orientation between individual chains to consider, which will in turn influence the residues that make up the interface. In consideration of this, CASP assessors specified three separate sets of scores; one for scoring the global fold, one indicating the global quality of the interface and a set of individual interface residue confidence scores which, in many ways, mirrored the Critical Assessment of PRediction of Interactions (CAPRI) scoring criteria where three scores (fnat, L‐rms and I‐rms^18^) are also used. The effect of this, from a predictor's point of view, is the intrinsic difficulty in identifying which target functions we should be optimizing our quality scores for. We developed the ModFOLDdock variants with the assessors' formulae for global and local scores in mind. However, this added to the complexity of the methodology, as each variant relies on the contribution of different scores to address each of the different facets of the quality estimation problem. As mentioned above, CAPRI use a standard measure for interface residues, but this is not completely consistent with the range of confidence scores required in the CASP15 EMA process.

Individual chain orientation within a multimer has a sizeable influence on the accuracy of the scores produced. This is especially true for clustering‐based methods, as the correct orientation of the individual chains will be assumed from the mean of the largest clusters of models and is thus a function of the quality and variety of the modeling software used. With increasing reliance on an AlphaFold2 (or similar deep learning methods) at the heart of many modeling tools, it is conceivable that, for a field flooded with very similar AlphaFold‐based models, consistent incorrectly predicted orientations could lead to erroneous estimates of accuracy, the models for targets with alternative conformations being a case in point.

Another influence on accuracy was the size of some structures, particularly H1111, H1114, T1115, and H1137, which created a real challenge for our server resource allocation. ModFOLDdock and ModFOLDdockR both perform all against all comparisons for distance calculations and therefore a large population of very large models will present a challenge in terms of both server load and processing time. As explained in Section 2.6, for these very large complexes of 1500 residues or more, it was impossible for us to carry out all‐against‐all pairwise comparisons within the 48 h window due to our CPU and RAM limitations (and our commitment to processing predictions for the other CASP15 categories). In these cases, for both the ModFOLDdock and ModFOLDdockR methods, we initially scored all models using voronota‐js‐voromqa thereafter selecting the top 40 models to act as the reference set for model comparisons. This shortcut may have led to some loss of information and potentially prediction accuracy.

ModFOLDdockS is designed to overcome some of the limitations of clustering, and it was more efficient as it used sets of 30 reference models from MultiFOLD, which were already pre‐calculated for each target in the initial regular round, against which the subsequent model comparisons could be made. However, this approach relies on our MultiFOLD modeling system that integrated two older versions of ColabFold,^19^ which also had memory constraints for very large targets.

Another area of concern was the problem of individual chain identification and mapping. Simply put, it is very difficult for a system to accurately and reliably compare many multi‐chain models if equivalent chains within the submitted models used different identifiers for different chains (e.g., A, B, C vs. C, B, A or even B, C, D etc.). Therefore, we had to make sure our server handled chain mapping problems, missing chain IDs, and out‐of‐sync chain IDs, standardizing them prior to scoring.

In future, we plan to further build on our approach by combining the quasi‐single model and pure single model approaches for scoring multimeric models, building on the successful approach we have used previously for monomeric models with our ModFOLD server. In this way, it should be possible to reduce the inaccuracies and inefficiencies that occur for scoring larger models through the optimal combination of a single‐model (pure and quasi) methods versus relying on clustering approaches.

Future versions of ModFOLDdock will also need to be reoptimized based on larger populations of more accurate multimer models for more targets, such as those scored in CASP15. Figure S6 shows how the performance of each individual scoring method compares with the combined scores used by each ModFOLDdock variant and new optimal score combinations based on the larger CASP15 multimer model dataset. While they are still competitive and, in most cases, they outperformed the individual component scores, the score combinations that were optimized for the CASP14 data are not optimal for the CASP15 models. Tables S3 and S4 show the performance data for every combination of the seven component scores in terms of correlations and ranking respectively based on the CASP15 model data. In future, we will also investigate combining the component scores using a simple NN, as we have done previously with our ModFOLD server.

Finally, the leading EMA groups were asked their opinion on whether anything was to be gained from a detailed knowledge of the modeling process. It is our conviction that quality estimates should be largely independent, so knowledge of the modeling process and self‐assessment should not influence or bias scores. An ideal QA method should not need to know anything about how the model was made and it should be able to produce accurate scores for any model regardless of the source.

## Supporting information


**DATA S1:** Supporting Information

## Data Availability

The data that supports the findings of this study are available in the supplementary material of this article and at https://predictioncenter.org/casp15.
