# Supplementary material for: Estimation of model accuracy in CASP15 using the ModFOLDdock server
Source: Proteins. 2023 Jun 14;91(12):1871–8. doi: 10.1002/prot.26532 (PMC10952711; doi:10.1002/prot.26532)
Supplement: Supplementary file 1 — DATA S1: Supporting Information [file PROT-91-1871-s001.docx]

**Supplementary Methods**

The ModFOLDIA method produces both global and per-residue interface scores. The first stage is to identify interface residues with <= 8Å between Cβ atoms (Cα for GLY) and calculate the minimum contact distance (*D_min_*) for each contacting residue. The second stage is to locate the equivalent residues in all other models and calculate the mean minimum distances across all other models (*MeanD_min_*). The final Interface Accuracy (IA) score for each interface residue in is the absolute difference in the *S_i_* from the mean *S_i_* : *IA = 1-|S_i_-MeanS_i_|*, where *S_i_ = 1/(1+(D_min_/20)^2^)* and *MeanS_i_ = 1/(1+(MeanD_min_/20)^2^)*. The global ModFOLDIA score for a model is the total interface score (sum of residue scores) normalised by the maximum of either the number of residues in the interface or the mean number of interface residues across all models for the same target.

The CDA score was based on the original Contact Distance Agreement (CDA) score^1,2^, which relates to the agreement between the residue contacts predicted from the sequence and the measured Euclidean distance (in Å) between residues in the model. In this case, we used the contact prediction profiles that resulted from the generation of LocalColabFold^3^ version 1.0.0 multimer models.

For the DockQJury, QSscoreJury, QSscoreOfficialJury and lDDTOfficialJury scoring methods, pairwise comparisons were made between each quaternary structure model and every other model and then the mean QS, lDDT or DockQ scores were calculated. The difference between the QSscoreJury and QSscoreOfficialJury approaches was that in the former, in-house code was used to calculate the fraction of correctly modelled interface contacts in the complex normalised by the max of either the observed or predicted contacts, while in the latter, the OpenStructure^4^ package was used to obtain QS scores (using the “ost compare-structures” action).

The voronota-js-voromqa method was used off-the-shelf with the “--inter-chain” and “--output-dark-scores” options.

1. McGuffin LJ, Shuid AN, Kempster R, Maghrabi AHA, Nealon JO, Salehe BR, Atkins JD, Roche DB. Accurate template-based modeling in CASP12 using the IntFOLD4-TS, ModFOLD6, and ReFOLD methods. Proteins 2018;86:335–344. doi: 10.1002/prot.25360

2. Maghrabi AHA and McGuffin LJ. ModFOLD6: an accurate web server for the global and local quality estimation of 3D protein models. Nucleic Acids Research 2017;45:W416-W421. doi:

3. Mirdita M, Schütze K, Moriwaki Y, Heo L, Ovchinnikov S, and Steinegger M. ColabFold: making protein folding accessible to all. Nature Methods 2022;19(6):679–682. doi: 10.1038/s41592-022-01488-1

4. Biasini M, Schmidt T, Bienert S, Mariani V, Studer G, Haas J, Johner N, Schenk AD, Philippsen A, Schwede T. OpenStructure: an integrated software framework for computational structural biology. Acta Crystallographica Section D: Biological Crystallography 2013;69,701–709. doi: 10.1107/S0907444913007051


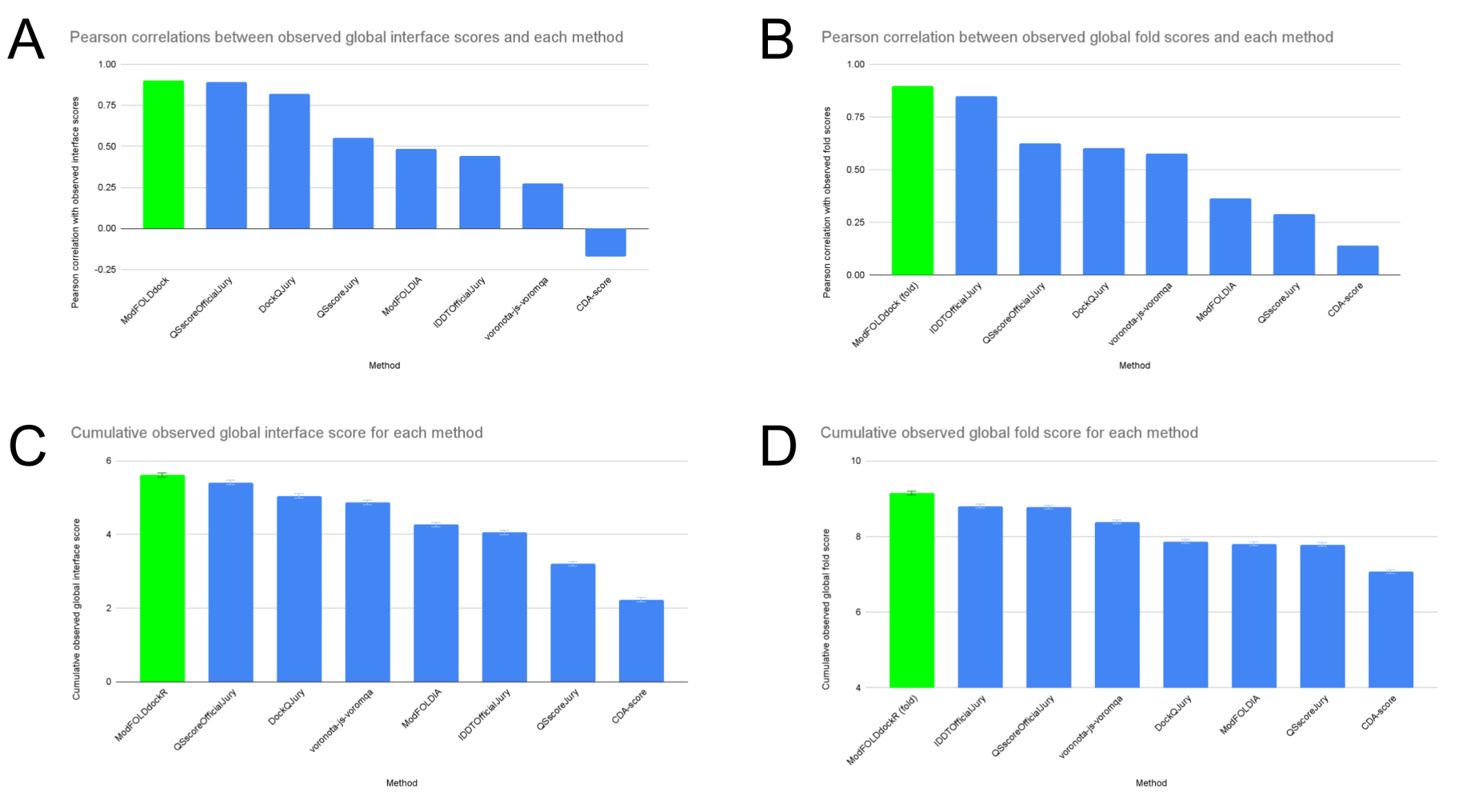


**Supplementary Figure 1.** Bar charts comparing the ModFOLDdock and ModFOLDdockR methods with their constituent scores using the CASP14 multimer data (2060 models). **A)** Pearson correlations between observed global interface scores (mean of F1 and Jaccard Coeff.) and each method. **B)** Pearson correlation between observed global fold scores (mean of Oligo-lDDT and TM-score) and each method. **C)** Cumulative observed global interface score for each method. **D)** Cumulative observed global fold score for each method.

**Supplementary Table 1.** Correlations between the observed global interface and fold scores and every combination of the 7 component scores, based on the CASP14 multimer data: A=ModFOLDIA, B=DockQJury, C=QSscoreJury, D=QSscoreOfficialJury, E=lDDTOfficialJury, F=voronota-js-voromqa, G=CDA-score. The table is sorted by the cumulative global fold score. The top scores in each column are shown in bold. The combinations used for the ModFOLDdock fold and interface scores are highlighted in green.

| Method combination | Interface | | | Fold | | |
| --- | --- | --- | --- | --- | --- | --- |
|  | Pearson | Spearman | Kendall | Pearson | Spearman | Kendall |
| B+E | 0.6221383 | 0.4662672 | 0.3370294 | **0.897708** | **0.8895329** | 0.7178826 |
| D+E | 0.7678932 | 0.6149145 | 0.451429 | 0.8886437 | 0.8864162 | **0.7204588** |
| B+D+E+F | 0.7370915 | 0.5618972 | 0.4084465 | 0.8755656 | 0.8648914 | 0.6910571 |
| D+E+F | 0.6796071 | 0.5390013 | 0.3894662 | 0.8748695 | 0.8658919 | 0.6912109 |
| B+D+E | 0.8155852 | 0.6325805 | 0.4671395 | 0.8738063 | 0.8812126 | 0.7138623 |
| B+E+F | 0.5398861 | 0.4028433 | 0.2887446 | 0.8507348 | 0.8403956 | 0.6561161 |
| E | 0.4398352 | 0.3730243 | 0.2678815 | 0.8503973 | 0.8587005 | 0.6726669 |
| E+F | 0.4053162 | 0.3287048 | 0.2352872 | 0.8024292 | 0.8084877 | 0.6153324 |
| B+C+D+E+F | 0.7941014 | 0.7417475 | 0.552793 | 0.7869698 | 0.7413835 | 0.564248 |
| C+D+E+F | 0.7561131 | 0.7344757 | 0.5432355 | 0.7773438 | 0.740087 | 0.5629397 |
| A+B+D+E+F | 0.7440054 | 0.7063684 | 0.5241025 | 0.7740935 | 0.7298914 | 0.5474411 |
| B+C+E+F | 0.6886405 | 0.682026 | 0.4928813 | 0.77257 | 0.7296966 | 0.5545109 |
| B+D+F | 0.82149 | 0.6114097 | 0.4479444 | 0.7606479 | 0.7381083 | 0.5590382 |
| D+F | 0.7698032 | 0.5881448 | 0.4268164 | 0.7599284 | 0.7393382 | 0.5587745 |
| A+D+E+F | 0.7021574 | 0.6944457 | 0.511267 | 0.7595768 | 0.7250281 | 0.5418011 |
| B+C+D+E | 0.8339838 | 0.7859983 | 0.592779 | 0.7462676 | 0.696275 | 0.520593 |
| C+E+F | 0.6134071 | 0.6547062 | 0.4648403 | 0.7417553 | 0.7143769 | 0.538481 |
| A+B+E+F | 0.6275222 | 0.629427 | 0.4553829 | 0.7412427 | 0.7066384 | 0.5248789 |
| A+B+D+E | 0.7705623 | 0.7432268 | 0.5556862 | 0.7353772 | 0.6776837 | 0.4981892 |
| C+D+E | 0.7973255 | 0.779981 | 0.5836362 | 0.7337487 | 0.6933543 | 0.5177601 |
| B+C+E | 0.7368397 | 0.7398483 | 0.5406193 | 0.7320624 | 0.6813849 | 0.5099464 |
| A+D+E | 0.7265591 | 0.7328193 | 0.5432948 | 0.7169642 | 0.671624 | 0.4915085 |
| B+F | 0.5660863 | 0.3635316 | 0.2592923 | 0.7137864 | 0.6852296 | 0.5128599 |
| B+D+E+F+G | 0.404772 | 0.3780916 | 0.2710916 | 0.7101008 | 0.7061886 | 0.553011 |
| A+B+C+D+E+F | 0.7635909 | 0.7739465 | 0.581026 | 0.7083849 | 0.6634288 | 0.4891847 |
| A+E+F | 0.5560334 | 0.5970161 | 0.4261682 | 0.7073137 | 0.6875881 | 0.5044643 |
| A+B+E | 0.6486699 | 0.6680471 | 0.4850158 | 0.6952704 | 0.6487127 | 0.4725049 |
| B+C+D+E+F+G | 0.5244234 | 0.5830563 | 0.412383 | 0.693544 | 0.6456508 | 0.4798443 |
| A+C+D+E+F | 0.7308335 | 0.7665216 | 0.5709852 | 0.6924358 | 0.6571411 | 0.4831878 |
| A+B+D+E+F+G | 0.5098543 | 0.5772571 | 0.4069296 | 0.6923436 | 0.6586154 | 0.4819218 |
| C+E | 0.651986 | 0.7111715 | 0.5072599 | 0.6921837 | 0.6585876 | 0.4896367 |
| A+B+C+E+F | 0.6820997 | 0.7336256 | 0.5382033 | 0.6785479 | 0.6422252 | 0.4708995 |
| D+E+F+G | 0.322943 | 0.336453 | 0.2426468 | 0.6655271 | 0.6797333 | 0.5371782 |
| A+B+C+D+E | 0.7767018 | 0.7949579 | 0.6008529 | 0.6636642 | 0.6161361 | 0.4468922 |
| A+B+C+D+E+F+G | 0.5752266 | 0.6696365 | 0.4772083 | 0.6628724 | 0.61441 | 0.4467918 |
| A+D+E+F+G | 0.4534166 | 0.5418677 | 0.3798655 | 0.6615978 | 0.6340081 | 0.4614429 |
| C+D+E+F+G | 0.4632582 | 0.5481547 | 0.3875863 | 0.6606479 | 0.6218252 | 0.4615751 |
| B+D+E+G | 0.3875972 | 0.3909207 | 0.2861465 | 0.657835 | 0.6568015 | 0.5137236 |
| A+E | 0.5664097 | 0.6327542 | 0.4510429 | 0.6517214 | 0.623084 | 0.4474086 |
| A+B+D+F | 0.7623274 | 0.7355312 | 0.5461741 | 0.6514716 | 0.5946109 | 0.4284181 |
| A+C+E+F | 0.6316307 | 0.7140192 | 0.5164196 | 0.650924 | 0.6241992 | 0.4542625 |
| A+B+D+E+G | 0.5031556 | 0.5929763 | 0.4203144 | 0.6474392 | 0.6030616 | 0.4331001 |
| B+C+D+E+G | 0.5184236 | 0.5958047 | 0.4257621 | 0.6438089 | 0.5959577 | 0.4371545 |
| A+C+D+E | 0.7419265 | 0.78715 | 0.5899385 | 0.6436479 | 0.6069291 | 0.4387054 |
| B+D | **0.9005487** | 0.8246907 | 0.6435966 | 0.6419381 | 0.5309702 | 0.3781203 |
| A+C+D+E+F+G | 0.5309632 | 0.6464253 | 0.4565751 | 0.6379261 | 0.5954371 | 0.4301802 |
| B+C+D+F | 0.8175272 | 0.7770315 | 0.5889212 | 0.637794 | 0.5740778 | 0.4180225 |
| D | 0.8904282 | **0.8440979** | **0.6601409** | 0.6263819 | 0.5468863 | 0.389032 |
| A+B+C+E | 0.692102 | 0.7551501 | 0.5565514 | 0.6252666 | 0.5871694 | 0.4235903 |
| A+D+F | 0.7117394 | 0.7213149 | 0.5301804 | 0.622492 | 0.5825022 | 0.4172562 |
| A+B+C+D+E+G | 0.5709968 | 0.6804326 | 0.4864792 | 0.6196486 | 0.5683172 | 0.406208 |
| A+B+E+F+G | 0.3599946 | 0.4367296 | 0.3031905 | 0.6129679 | 0.5757836 | 0.417113 |
| A+D+E+G | 0.4407902 | 0.5547524 | 0.3917973 | 0.6112724 | 0.5745229 | 0.4106077 |
| C+D+F | 0.7728359 | 0.7688163 | 0.5767856 | 0.6092083 | 0.5646974 | 0.4102714 |
| B+C+E+F+G | 0.3607219 | 0.4482979 | 0.3162082 | 0.6082477 | 0.5674795 | 0.4255933 |
| C+D+E+G | 0.4501244 | 0.5582474 | 0.4005675 | 0.6043174 | 0.5679783 | 0.4172322 |
| A+B+C+E+F+G | 0.4609644 | 0.5808734 | 0.4031719 | 0.6038874 | 0.5565473 | 0.3965046 |
| D+E+G | 0.2933906 | 0.3537209 | 0.2610584 | 0.6029764 | 0.6337003 | 0.5030273 |
| B | 0.8191334 | 0.6607223 | 0.508491 | 0.6028232 | 0.4526431 | 0.3333887 |
| A+B+D+F+G | 0.4890414 | 0.5849852 | 0.4139539 | 0.5929576 | 0.5377722 | 0.3810692 |
| A+C+E | 0.6362988 | 0.7327199 | 0.5304996 | 0.5909937 | 0.5620175 | 0.402464 |
| A+C+D+E+G | 0.5230199 | 0.6551545 | 0.4642964 | 0.5908642 | 0.5464175 | 0.3876725 |
| B+D+F+G | 0.354711 | 0.3933636 | 0.2905136 | 0.590493 | 0.5839326 | 0.4563542 |
| A+B+C+D+F | 0.7620261 | 0.790109 | 0.5947494 | 0.5876953 | 0.5479381 | 0.3910265 |
| B+E+F+G | 0.1851618 | 0.2087258 | 0.1507441 | 0.5831396 | 0.6225731 | 0.4928905 |
| A+B+F | 0.618582 | 0.6458525 | 0.4640224 | 0.5803044 | 0.5419518 | 0.3857262 |
| B+C+D+F+G | 0.5012339 | 0.5883603 | 0.4232738 | 0.5783218 | 0.5269261 | 0.3826374 |
| F | 0.2763438 | 0.1935796 | 0.1387928 | 0.5760358 | 0.5914943 | 0.4220089 |
| B+C+F | 0.693108 | 0.7096416 | 0.5203948 | 0.5736845 | 0.5263652 | 0.383877 |
| A+C+E+F+G | 0.4048945 | 0.5446632 | 0.3756973 | 0.5690548 | 0.5268085 | 0.3730967 |
| A+E+F+G | 0.2891742 | 0.391625 | 0.271775 | 0.5683542 | 0.5419194 | 0.3920983 |
| A+B+C+D+F+G | 0.5594844 | 0.674622 | 0.482897 | 0.5664158 | 0.5174481 | 0.3660899 |
| A+B+D | 0.768613 | 0.7525183 | 0.558776 | 0.5610124 | 0.4917038 | 0.3451411 |
| A+C+D+F | 0.7226791 | 0.7797477 | 0.5814859 | 0.5604365 | 0.5346216 | 0.380508 |
| C+E+F+G | 0.2814387 | 0.4114624 | 0.2892574 | 0.5582347 | 0.5390696 | 0.4061662 |
| A+B+E+G | 0.3378762 | 0.4456156 | 0.3136935 | 0.5549658 | 0.511629 | 0.3660532 |
| A+B+C+E+G | 0.4475483 | 0.5869245 | 0.410364 | 0.5516895 | 0.5016167 | 0.3515447 |
| A+D+F+G | 0.4182374 | 0.5407973 | 0.3815873 | 0.5494807 | 0.5019142 | 0.3543656 |
| B+C+E+G | 0.3358963 | 0.4615773 | 0.3322916 | 0.5424231 | 0.5047689 | 0.379338 |
| A+C+D+F+G | 0.5065592 | 0.6472278 | 0.4586581 | 0.5327191 | 0.491706 | 0.3455039 |
| C+D+F+G | 0.4227912 | 0.5436172 | 0.3943254 | 0.5296769 | 0.4914799 | 0.359338 |
| A+B+C+F | 0.6645752 | 0.741465 | 0.5427793 | 0.5288693 | 0.5045896 | 0.3578339 |
| B+C+D | 0.8269361 | 0.8091608 | 0.6240246 | 0.5266822 | 0.4474464 | 0.3143817 |
| D+F+G | 0.2409491 | 0.3563423 | 0.2642462 | 0.5200666 | 0.5632211 | 0.4457454 |
| A+D | 0.7113254 | 0.7359118 | 0.5398023 | 0.5183876 | 0.472099 | 0.3289444 |
| A+F | 0.5191631 | 0.5945452 | 0.416958 | 0.5173752 | 0.4959425 | 0.3466355 |
| E+F+G | 0.08656197 | 0.1623278 | 0.1190342 | 0.516321 | 0.5948857 | 0.4702613 |
| A+B+D+G | 0.4680578 | 0.5842217 | 0.4176302 | 0.5161636 | 0.4451738 | 0.3088823 |
| A+C+E+G | 0.3863691 | 0.5462825 | 0.3798923 | 0.5119406 | 0.4660067 | 0.3253892 |
| A+B+C+D | 0.7580323 | 0.7981737 | 0.6005767 | 0.5110592 | 0.4718043 | 0.3301004 |
| B+E+G | 0.1361889 | 0.2239605 | 0.1650852 | 0.5046949 | 0.5940821 | 0.4727034 |
| A+E+G | 0.2593274 | 0.3971522 | 0.2793416 | 0.5032234 | 0.4725844 | 0.338792 |
| A+B+C+D+G | 0.5443057 | 0.6752321 | 0.4848112 | 0.5008184 | 0.4516605 | 0.3133573 |
| C+F | 0.5830781 | 0.6636917 | 0.4721933 | 0.5005905 | 0.4783326 | 0.347197 |
| B+C+D+G | 0.4768018 | 0.5843225 | 0.4297474 | 0.4884084 | 0.4384612 | 0.3118733 |
| A+B+C+F+G | 0.4226695 | 0.5722815 | 0.4006841 | 0.4856252 | 0.4384016 | 0.3041032 |
| A+C+F | 0.600404 | 0.7118308 | 0.5111797 | 0.4840006 | 0.4705569 | 0.331071 |
| C+E+G | 0.2468374 | 0.4239733 | 0.303394 | 0.483393 | 0.4721439 | 0.3579832 |
| A+B+F+G | 0.3005459 | 0.423398 | 0.2983356 | 0.4808792 | 0.4279865 | 0.3033854 |
| C+D | 0.776152 | 0.8001199 | 0.6106079 | 0.4783409 | 0.430884 | 0.301257 |
| B+D+G | 0.3094441 | 0.4280989 | 0.3231873 | 0.4782347 | 0.476807 | 0.362241 |
| A+C+D | 0.7141838 | 0.7852169 | 0.5846252 | 0.4757161 | 0.4529065 | 0.3157709 |
| A+D+G | 0.3891704 | 0.5360537 | 0.3833224 | 0.4642765 | 0.4031501 | 0.2789366 |
| A+C+D+G | 0.4865639 | 0.6439374 | 0.4579553 | 0.46168 | 0.4198982 | 0.2893849 |
| A+B | 0.6071178 | 0.6558811 | 0.4686983 | 0.4534903 | 0.4125144 | 0.285917 |
| B+C+F+G | 0.2905314 | 0.4458545 | 0.3235596 | 0.4523077 | 0.4241992 | 0.318057 |
| A+C+F+G | 0.3548218 | 0.5249093 | 0.3653983 | 0.439687 | 0.3948 | 0.2728716 |
| A+B+C | 0.650793 | 0.7447487 | 0.5436193 | 0.4311374 | 0.4121513 | 0.2869405 |
| C+D+G | 0.3883645 | 0.5329598 | 0.3977753 | 0.429081 | 0.3939667 | 0.2836269 |
| E+G | 0.02468937 | 0.1761515 | 0.1302842 | 0.4251367 | 0.5724286 | 0.4517576 |
| A+F+G | 0.2113104 | 0.3688911 | 0.2583106 | 0.4196708 | 0.3812478 | 0.2700233 |
| A+B+C+G | 0.3957246 | 0.5623156 | 0.3984426 | 0.4068951 | 0.3560086 | 0.2435724 |
| B+C | 0.6909383 | 0.7484381 | 0.5628754 | 0.4061099 | 0.3721297 | 0.2628535 |
| B+F+G | 0.05367426 | 0.1978878 | 0.1437184 | 0.3952618 | 0.5245636 | 0.4094789 |
| D+G | 0.1774821 | 0.4080011 | 0.306333 | 0.3898023 | 0.4662864 | 0.3551107 |
| A+B+G | 0.2594555 | 0.4228055 | 0.3027759 | 0.3843279 | 0.32582 | 0.2272073 |
| C+F+G | 0.1884297 | 0.405049 | 0.2890516 | 0.3814986 | 0.385034 | 0.291479 |
| A+C | 0.5777453 | 0.7109297 | 0.5076395 | 0.3745237 | 0.3699924 | 0.2545747 |
| A | 0.4867195 | 0.5868364 | 0.4057687 | 0.3654596 | 0.3366946 | 0.2293649 |
| A+C+G | 0.3220312 | 0.5120821 | 0.3609473 | 0.3550122 | 0.307833 | 0.2099138 |
| B+C+G | 0.241169 | 0.4606827 | 0.3420289 | 0.3371482 | 0.3392478 | 0.250484 |
| A+G | 0.1615911 | 0.3670324 | 0.2590266 | 0.3145923 | 0.2748015 | 0.1898057 |
| F+G | -0.07501003 | 0.1258351 | 0.0909146 | 0.2987003 | 0.4859893 | 0.3728236 |
| C | 0.5505007 | 0.6904874 | 0.5030818 | 0.2886947 | 0.2966009 | 0.2073917 |
| C+G | 0.128631 | 0.4234011 | 0.3054489 | 0.2560357 | 0.2986689 | 0.2202136 |
| B+G | -0.03138041 | 0.2517043 | 0.181093 | 0.2454451 | 0.4397153 | 0.3312597 |
| G | -0.1693019 | 0.1327867 | 0.09281258 | 0.1382419 | 0.3581771 | 0.2661346 |

**Supplementary Table 2.** Cumulative observed global interface and fold scores of the top raked models for every combination of the 7 component scores based on the CASP14 multimer data: A=ModFOLDIA, B=DockQJury, C=QSscoreJury, D=QSscoreOfficialJury, E=lDDTOfficialJury, F=voronota-js-voromqa, G=CDA-score. The table is sorted by the cumulative global fold score. The top scores in each column are shown in bold. The ModFOLDdockR fold and interface score combinations are highlighted in green.

| Method combination | Interface | Fold |
| --- | --- | --- |
| **C+E+F** | 4.962 | **9.145** |
| B+D+G | 5.2505 | 9.097 |
| E+F | 5.04 | 9.091 |
| B+E+F | 5.4545 | 9.0885 |
| D+E+F | 5.117 | 9.064 |
| B+E+F+G | 5.136 | 9.0625 |
| C+D+E+G | 5.006 | 9.0485 |
| B+E | 5.167 | 9.01 |
| D+E | 5.3215 | 9.003 |
| B+C+D+E+G | 5.196 | 8.9935 |
| B+C+D+E | 5.2155 | 8.985 |
| A+B+C+D+E+F+G | 5.0855 | 8.956 |
| B+D+E | 5.345 | 8.948 |
| B+C+D+G | 5.126 | 8.9285 |
| B+D+E+F | 5.1455 | 8.913 |
| D+E+G | 4.8725 | 8.9055 |
| A+C+F | 5.286 | 8.883 |
| D+E+F+G | 4.6635 | 8.8575 |
| C+D+E | 4.919 | 8.856 |
| C+D+G | 4.6205 | 8.8535 |
| B+D+E+G | 4.912 | 8.812 |
| A+B+D+E+F+G | 4.944 | 8.8085 |
| E | 4.0515 | 8.805 |
| B+D+E+F+G | 4.657 | 8.802 |
| A+B+D+E+F | 5.0655 | 8.797 |
| A+B+C+D+F+G | 5.2595 | 8.785 |
| A+B+C+D+E+G | 4.931 | 8.7825 |
| A+C+D+E+G | 4.931 | 8.7825 |
| B+C+E+F | 4.7155 | 8.7825 |
| A+B+C+E | 5.3425 | 8.7795 |
| D | 5.414 | 8.7745 |
| A+B+C+D+E+F | 5.408 | 8.7745 |
| C+D+E+F | 4.6355 | 8.774 |
| D+G | 4.6135 | 8.7625 |
| A+C+D+E+F+G | 4.9075 | 8.7585 |
| A+B+D+F | 5.3465 | 8.7565 |
| A+B+D+F+G | 4.905 | 8.7535 |
| A+B+C+E+F | 4.8465 | 8.7495 |
| A+B+C+E+G | 4.757 | 8.7145 |
| A+B+C+E+F+G | 4.7275 | 8.7075 |
| B+D+F+G | 4.8505 | 8.707 |
| B+C+D+E+F | 4.943 | 8.704 |
| A+B+F+G | 4.6465 | 8.6965 |
| C+E+F+G | 4.3265 | 8.6965 |
| A+C+D+E | 5.114 | 8.68 |
| B+E+G | 4.3635 | 8.6705 |
| A+D+E+F | 4.7285 | 8.67 |
| B+C+E+G | 4.339 | 8.67 |
| E+F+G | 4.349 | 8.6685 |
| A+B+C+F+G | 4.4715 | 8.6675 |
| A+B+C+D+F | 5.335 | 8.666 |
| A+D+E+F+G | 4.6845 | 8.6655 |
| A+C+D+E+F | 4.7645 | 8.6605 |
| A+B+E+F+G | 4.646 | 8.6535 |
| A+B+E+F | 4.5695 | 8.6525 |
| A+C+F+G | 4.7275 | 8.648 |
| A+B+C+F | 5.057 | 8.6475 |
| A+D+F | 5.207 | 8.6465 |
| C+D+F+G | 4.5735 | 8.646 |
| A+B+C+D+E | 5.1365 | 8.644 |
| B+C+D+F+G | 4.796 | 8.6435 |
| A+C+E+F | 4.6035 | 8.6425 |
| C+D+E+F+G | 4.5855 | 8.6385 |
| B+C+D+F | 5.4965 | 8.638 |
| C+D+F | 5.185 | 8.638 |
| B+C+D+E+F+G | 4.808 | 8.636 |
| A+B+D+G | 5.238 | 8.6305 |
| A+D+E+G | 4.7865 | 8.6305 |
| A+B+D+E+G | 4.776 | 8.6185 |
| A+F+G | 4.4245 | 8.615 |
| A+C+E | 4.87 | 8.6145 |
| B+F+G | 4.852 | 8.602 |
| A+C+E+F+G | 4.558 | 8.5985 |
| C+E | 4.2275 | 8.596 |
| A+D+F+G | 4.7225 | 8.5825 |
| A+C+D | 5.2855 | 8.5745 |
| C+F+G | 3.9805 | 8.574 |
| B+C+E+F+G | 4.559 | 8.572 |
| A+D+E | 4.9065 | 8.57 |
| B+C+F+G | 4.549 | 8.5665 |
| D+F | 5.4435 | 8.564 |
| D+F+G | 4.687 | 8.563 |
| C+E+G | 4.036 | 8.555 |
| A+C+D+F+G | 4.9095 | 8.5535 |
| A+D+G | 5.1505 | 8.5475 |
| B+D | 5.412 | 8.547 |
| B+C+F | 5.3875 | 8.5365 |
| A+C | 5.247 | 8.5345 |
| B+C+G | 4.3575 | 8.5335 |
| A+B+E+G | 4.365 | 8.5065 |
| A+B+D+E | 4.8725 | 8.493 |
| A+B+C+D+G | 5.4715 | 8.488 |
| A+B+E | 4.6945 | 8.4835 |
| A+C+D+G | 5.283 | 8.483 |
| **B+D+F** | **5.6105** | 8.479 |
| A+C+E+G | 4.394 | 8.4625 |
| B+C+E | 4.4885 | 8.4585 |
| A+D | 5.206 | 8.448 |
| A+B+C+D | 5.478 | 8.4325 |
| A+C+D+F | 4.8075 | 8.42 |
| A+C+G | 4.776 | 8.418 |
| B+C+D | 5.2495 | 8.4175 |
| A+B+G | 4.775 | 8.4065 |
| A+E | 4.6635 | 8.405 |
| C+F | 4.6335 | 8.403 |
| F | 4.8695 | 8.3865 |
| A+B+F | 4.592 | 8.3765 |
| A+B+C | 5.026 | 8.367 |
| A+B+D | 5.286 | 8.3585 |
| A+F | 4.522 | 8.3505 |
| A+E+G | 4.146 | 8.3485 |
| A+E+F+G | 4.2285 | 8.3415 |
| A+B+C+G | 5.02 | 8.3305 |
| C+G | 3.3645 | 8.315 |
| C+D | 4.6965 | 8.3065 |
| B+F | 5.449 | 8.2965 |
| A+G | 4.562 | 8.2915 |
| A+E+F | 4.0765 | 8.207 |
| E+G | 2.579 | 8.166 |
| F+G | 3.4355 | 8.112 |
| A+B | 4.8615 | 8.089 |
| B+G | 3.818 | 8.055 |
| B+C | 4.4975 | 7.964 |
| B | 5.048 | 7.8685 |
| A | 4.265 | 7.808 |
| C | 3.201 | 7.788 |
| G | 2.2295 | 7.0715 |


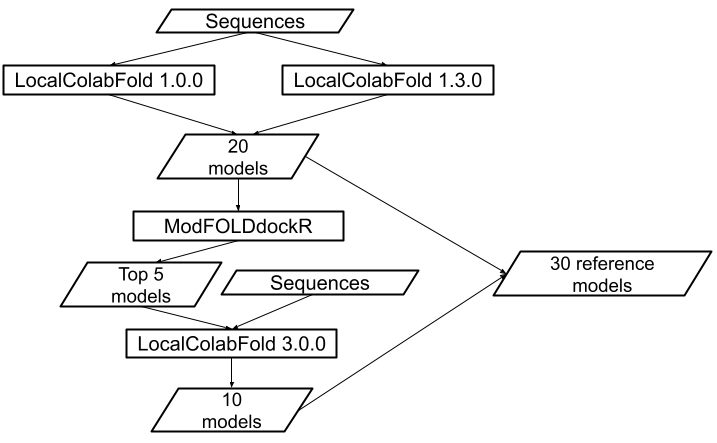


**Supplementary Figure 2.** The MultiFOLD modelling pathway developed by the McGuffin group and used at CASP15, which includes a variant of ModFOLDdock to rank models prior to the recycling step.


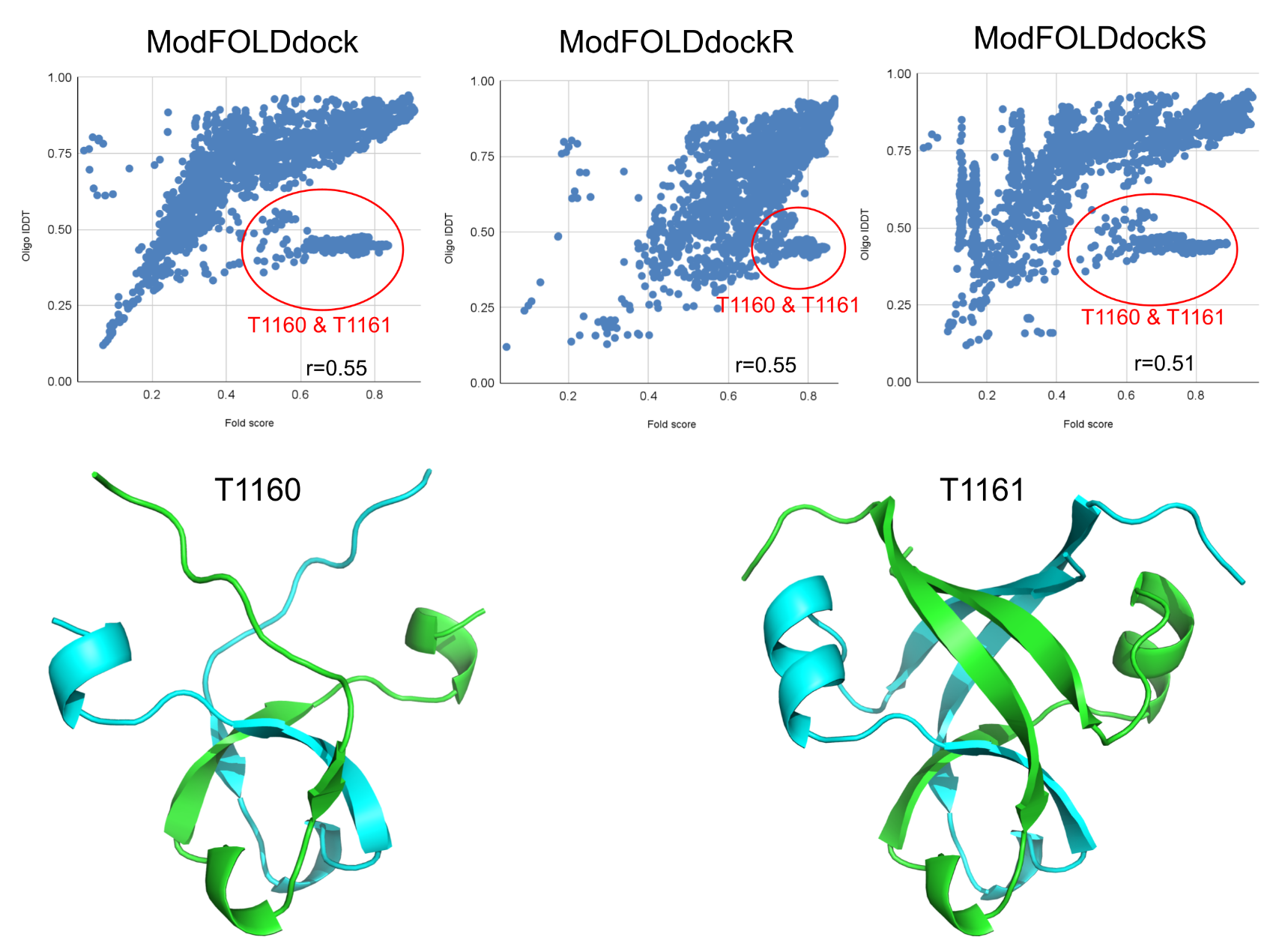


**Supplementary Figure 3.** Scatter plots and Pearson R values for each ModFOLDdock variant showing the predicted global fold scores (x-axis) versus the oligo-lDDT scores (y-axis) for all *homomeric* targets (upper panel) including the outlier models for T1160 and T1161 (lower panel).


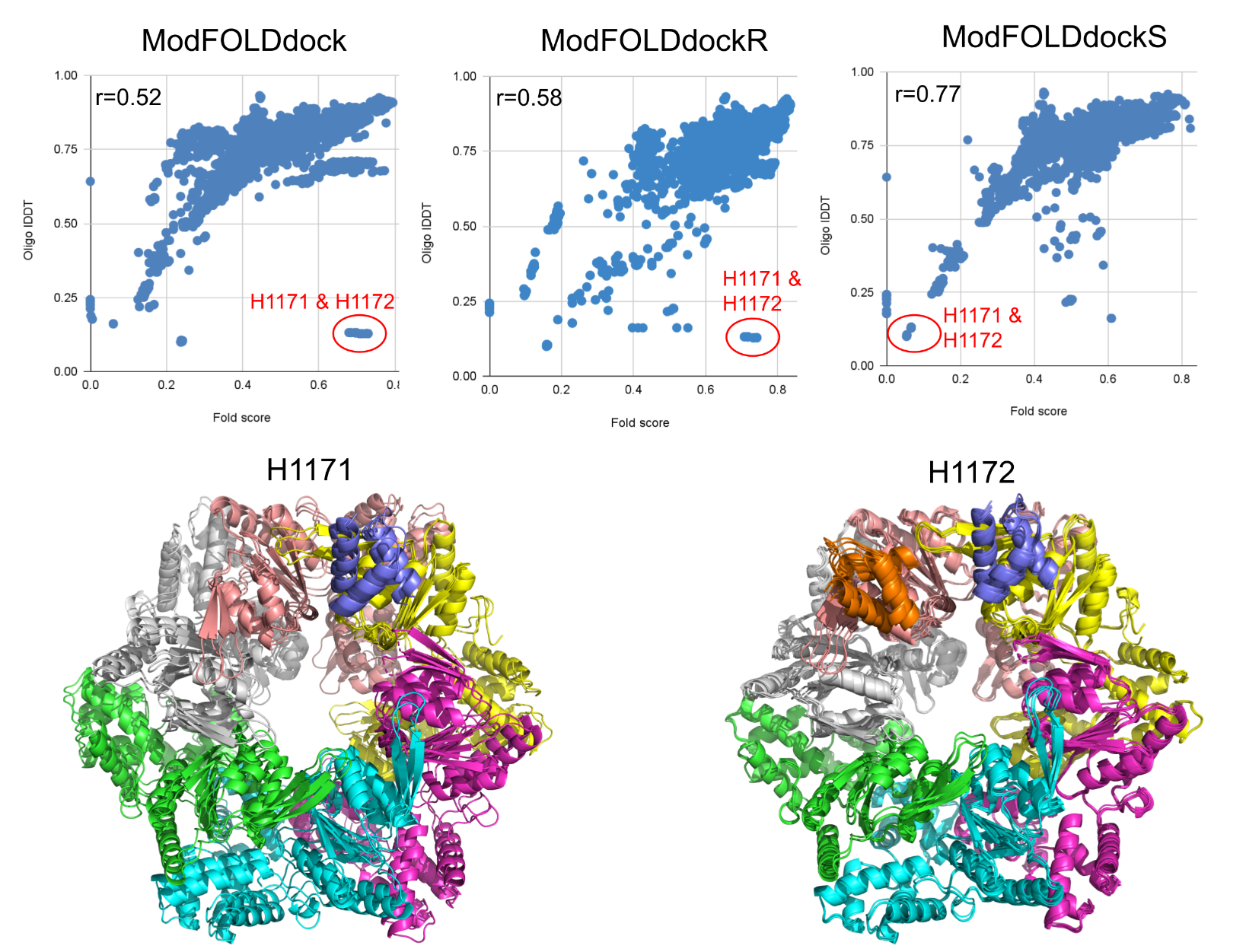


**Supplementary Figure 4.** Scatter plots and Pearson R figures for each ModFOLDdock variant showing the predicted global fold scores (x-axis) versus the oligo-lDDT scores (y-axis) for all *heteromeric* targets (upper panel) including the outlier models for H1171 and H1172 (lower panel).


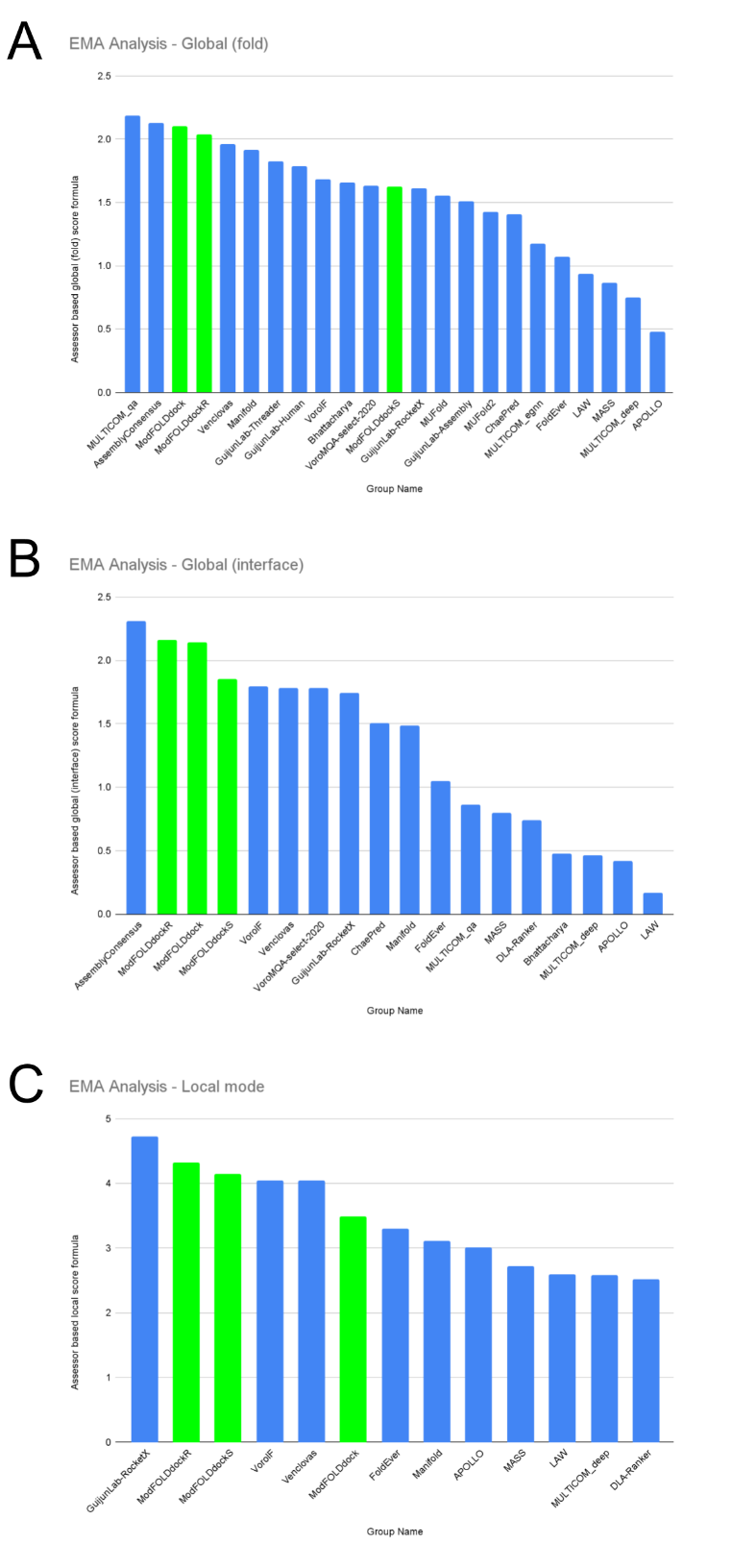


**Supplementary Figure 5.** Bar charts using data from the official CASP15 EMA evaluation of predicted versus observed model quality scores. Raw score data are from <https://predictioncenter.org/casp15/qa_global_fold.cgi>. The “Assessor based” formulae produce similar rankings to the official ones but using all raw scores: <https://predictioncenter.org/casp15/zscores_EMA.cgi>. **A)** Global fold “Assessor based formula” calculated as: (0.5*Pearson(GDT_TS))+(0.5*Spearman(GDT_TS))+AUC(GDT_TS)-Loss(GDT_TS)+ (0.5*Pearson(TM))+(0.5*Spearman(TM))+AUC(TM)-Loss(TM). **B)** Global interface score “Assessor based formula” calculated as: (0.5*Pearson(DockQ-wave))+(0.5*Spearman(DockQ-wave))+AUC(DockQ-wave)-Loss(DockQ-wave)+ (0.5*Pearson(QS))+(0.5*Spearman(QS))+AUC(QS)-Loss(QS). **C)** Local mode score “Assessor based formula” calculated as: (0.5*Pearson(PatchDockQ))+(0.5*Spearman(PatchDockQ))+AUC(PatchDockQ)+(0.5*Pearson(PatchQS))+(0.5*Spearman(PatchQS))+AUC(PatchQS)+(0.5*Pearson(CAD))+(0.5*Spearman(CAD))+AUC(CAD)+(0.5*Pearson(lDDT))+(0.5*Spearman(lDDT))+AUC(lDDT).


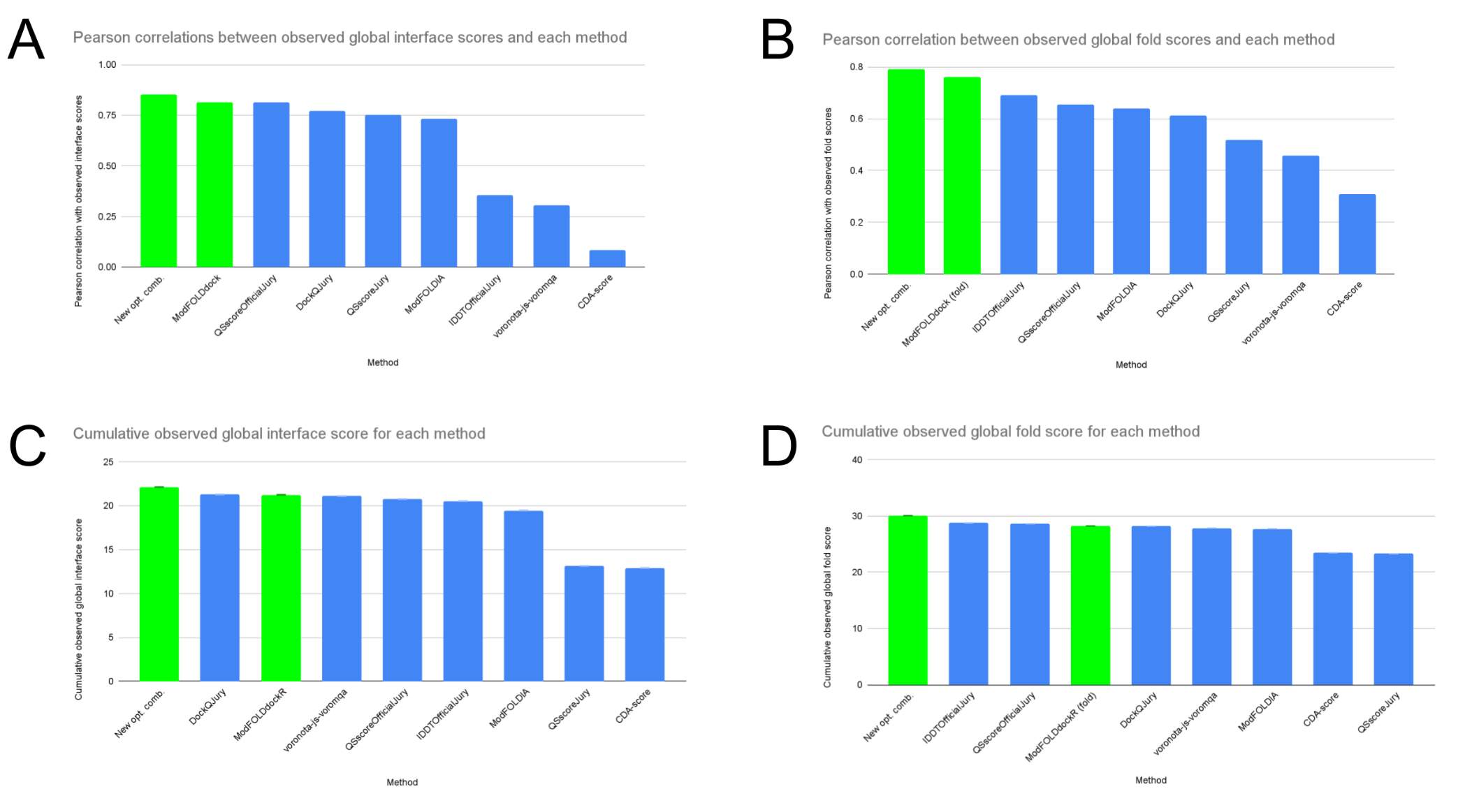


**Supplementary Figure 6.** Bar charts comparing the ModFOLDdock and ModFOLDdockR methods with their constituent scoring methods and the new optimal combinations (New opt. comb., see Supplementary Tables 3 and 4), using the CASP15 multimer data (10,041 models). A) Pearson correlations between observed global interface scores and each method. B) Pearson correlation between observed global fold scores and each method. C) Cumulative observed global interface score for each method. D) Cumulative observed global fold score for each method.

**Supplementary Table 3.** Correlations between the observed global interface and fold scores and every combination of the 7 component scores, based on the CASP15 multimer data: A=ModFOLDIA, B=DockQJury, C=QSscoreJury, D=QSscoreOfficialJury, E=lDDTOfficialJury, F=voronota-js-voromqa, G=CDA-score. The table is sorted by the cumulative global fold score. The top scores in each column are shown in bold. The combinations used for the ModFOLDdock fold and interface scores are highlighted in green.

| Method combination | Interface | | | Fold | | |
| --- | --- | --- | --- | --- | --- | --- |
|  | Pearson | Spearman | Kendall | Pearson | Spearman | Kendall |
| **A+B+E+F** | 0.7607744 | 0.7810566 | 0.5863143 | **0.792197** | 0.8110508 | 0.6264871 |
| **A+B+E** | 0.7835976 | 0.7941731 | 0.6018499 | 0.7920167 | **0.8124656** | 0.6288465 |
| A+E | 0.6712423 | 0.724254 | 0.5341655 | 0.7907918 | 0.7580845 | 0.5726135 |
| A+D+E+F | 0.7631311 | 0.778302 | 0.5864654 | 0.7828798 | 0.7810397 | 0.5917555 |
| A+D+E | 0.7817571 | 0.7880757 | 0.5992248 | 0.779598 | 0.7794885 | 0.5906576 |
| A+E+F | 0.6427604 | 0.6953987 | 0.5062022 | 0.7792268 | 0.7494083 | 0.5605032 |
| A+B+D+E+F | 0.7999895 | 0.8013594 | 0.6109345 | 0.77024 | 0.8001577 | 0.6103654 |
| B+E+F | 0.6700245 | 0.6832315 | 0.4922634 | 0.7662424 | 0.8041634 | 0.6214411 |
| D+E+F | 0.691555 | 0.710444 | 0.51523 | 0.7651328 | 0.7756342 | 0.5913521 |
| A+B+D+E | 0.8123319 | 0.8094355 | 0.6224629 | 0.7632537 | 0.7992152 | 0.6095188 |
| A+B+D+E+F+G | 0.71213 | 0.7254628 | 0.537127 | 0.7597706 | 0.7735538 | 0.5877849 |
| B+E | 0.6881144 | 0.6947989 | 0.5021146 | 0.7594839 | 0.8111374 | **0.6325391** |
| D+E | 0.7073283 | 0.7217248 | 0.5278964 | 0.7560278 | 0.778837 | 0.5952305 |
| A+B+C+E+F | 0.796953 | 0.8097918 | 0.6218742 | 0.7552738 | 0.7859826 | 0.595993 |
| A+B+C+E+F+G | 0.7103322 | 0.7340716 | 0.5488789 | 0.7548973 | 0.7637031 | 0.5778891 |
| A+B+D+E+G | 0.7174583 | 0.7299804 | 0.5412502 | 0.7525258 | 0.7725529 | 0.5866742 |
| A+B+C+D+E+F+G | 0.7552234 | 0.7654945 | 0.5791477 | 0.7521324 | 0.7733449 | 0.5843547 |
| A+B+E+F+G | 0.6317661 | 0.6624518 | 0.4818118 | 0.7520429 | 0.7473438 | 0.5681785 |
| A+B+C+E | 0.8147187 | 0.817736 | 0.6334889 | 0.750552 | 0.7847875 | 0.5947564 |
| B+D+E+F | 0.7554558 | 0.7527108 | 0.5562461 | 0.750184 | 0.7989597 | 0.6125388 |
| A+B+C+E+G | 0.7192154 | 0.7406937 | 0.5551352 | 0.7498999 | 0.7635227 | 0.577747 |
| A+C+D+E+F | 0.7937351 | 0.8028709 | 0.6174595 | 0.7479202 | 0.7628115 | 0.5696923 |
| A+C+E+F | 0.7378464 | 0.778311 | 0.587912 | 0.7478943 | 0.7458184 | 0.5568614 |
| A+C+E | 0.7628461 | 0.7901241 | 0.6027966 | 0.7472925 | 0.7452134 | 0.5580874 |
| A+C+D+E+F+G | 0.710417 | 0.7378565 | 0.5506054 | 0.7461249 | 0.7458769 | 0.5590313 |
| A+B+C+D+E+G | 0.7620347 | 0.7707219 | 0.5846652 | 0.7460696 | 0.7726765 | 0.5834702 |
| A+D+E+F+G | 0.6407502 | 0.6745225 | 0.4891441 | 0.745441 | 0.7307951 | 0.5487733 |
| A+B+E+G | 0.6359304 | 0.6631588 | 0.4824462 | 0.7445995 | 0.7440008 | 0.5650839 |
| A+B+C+D+E+F | 0.8171049 | 0.8155147 | 0.6302521 | 0.74337 | 0.7797262 | 0.5863781 |
| A+C+D+E | 0.8085262 | 0.8089784 | 0.6262927 | 0.7417401 | 0.7598942 | 0.5670028 |
| A+C+D+E+G | 0.7177058 | 0.743809 | 0.5561438 | 0.7399429 | 0.7446379 | 0.5583895 |
| B+C+E+F | 0.7550783 | 0.7717568 | 0.5768074 | 0.7372922 | 0.7828802 | 0.593157 |
| B+D+E | 0.7651851 | 0.7605444 | 0.5668158 | 0.7371088 | 0.8005139 | 0.614437 |
| A+D+E+G | 0.6441665 | 0.6774944 | 0.4913847 | 0.7369774 | 0.727735 | 0.5465505 |
| A+B+C+D+E | 0.8275648 | 0.8214247 | 0.6391157 | 0.7360316 | 0.7775514 | 0.5839635 |
| C+D+E+F | 0.7574503 | 0.7719266 | 0.5798163 | 0.7321345 | 0.7580168 | 0.5647637 |
| A+B+D+F+G | 0.7504968 | 0.75056 | 0.5623138 | 0.7304385 | 0.7587221 | 0.5702018 |
| B+C+E | 0.7739835 | 0.7824024 | 0.5914458 | 0.7283892 | 0.781302 | 0.5919408 |
| B+C+D+E+F+G | 0.7095713 | 0.7218773 | 0.5319105 | 0.7279556 | 0.7617822 | 0.5736333 |
| A+C+E+F+G | 0.6215696 | 0.6683992 | 0.4876848 | 0.7278256 | 0.7032684 | 0.5226722 |
| B+C+D+E+F | 0.790224 | 0.7891518 | 0.5969888 | 0.7263446 | 0.7761655 | 0.5827882 |
| C+E+F | 0.6532957 | 0.7254225 | 0.5289816 | 0.7223998 | 0.7466946 | 0.5561791 |
| B+D+E+F+G | 0.6398742 | 0.6528045 | 0.4686671 | 0.7221926 | 0.7513554 | 0.5689218 |
| C+D+E | 0.7727616 | 0.7806327 | 0.5918387 | 0.7217262 | 0.7556415 | 0.5617195 |
| A+B+C+D+F+G | 0.7883344 | 0.7870553 | 0.6029026 | 0.7215981 | 0.75715 | 0.5658271 |
| A+D+F | 0.8208233 | 0.8202029 | 0.6327292 | 0.7215653 | 0.7433735 | 0.5486325 |
| A+C+E+G | 0.6277953 | 0.6734435 | 0.49235 | 0.721509 | 0.7013592 | 0.5230312 |
| C+E | 0.6817425 | 0.7471613 | 0.5538573 | 0.721213 | 0.7504342 | 0.5608581 |
| A+B+C+F+G | 0.750662 | 0.7576033 | 0.5737377 | 0.7193098 | 0.7427258 | 0.5562919 |
| A+B+F | 0.8158347 | 0.8304724 | 0.6398981 | 0.7184514 | 0.7676853 | 0.575242 |
| B+C+D+E+G | 0.7137771 | 0.7273061 | 0.5380214 | 0.718074 | 0.7605741 | 0.5722769 |
| A+B+D+G | 0.7562067 | 0.7551106 | 0.5662869 | 0.7175999 | 0.7554038 | 0.5665718 |
| B+C+E+F+G | 0.6342171 | 0.6612457 | 0.47889 | 0.7174932 | 0.7380675 | 0.554694 |
| A+B+D+F | 0.8396594 | 0.8367958 | 0.6503316 | 0.7164302 | 0.7696049 | 0.5737324 |
| A+B+F+G | 0.6719427 | 0.685781 | 0.5040823 | 0.7159469 | 0.721086 | 0.5403913 |
| B+C+D+E | 0.7999324 | 0.7956452 | 0.6068064 | 0.7149956 | 0.7734081 | 0.5796942 |
| A+C+D+F+G | 0.7487798 | 0.7568664 | 0.5709277 | 0.7126201 | 0.7229114 | 0.5373882 |
| A+D+F+G | 0.6810514 | 0.694587 | 0.5094902 | 0.7126134 | 0.7049864 | 0.523308 |
| C+D+E+F+G | 0.6423852 | 0.6744323 | 0.4885018 | 0.7123693 | 0.7221012 | 0.5371319 |
| A+B+C+D+G | 0.7955141 | 0.7931492 | 0.6096297 | 0.7112625 | 0.7548669 | 0.5633322 |
| B+D+E+G | 0.63943 | 0.6500625 | 0.4671564 | 0.7084661 | 0.7485981 | 0.5661293 |
| A+B+C+G | 0.7612132 | 0.7656908 | 0.5808425 | 0.7081616 | 0.7400835 | 0.5536345 |
| B+C+E+G | 0.6374592 | 0.6625329 | 0.4813985 | 0.7061952 | 0.7352544 | 0.5524835 |
| A+D | 0.841963 | 0.8304054 | 0.645904 | 0.7032263 | 0.7346143 | 0.5397216 |
| A+C+D+G | 0.7571996 | 0.7630838 | 0.5756626 | 0.7008088 | 0.7200673 | 0.5346703 |
| C+D+E+G | 0.6448817 | 0.6793637 | 0.4937322 | 0.7005208 | 0.7203016 | 0.5361504 |
| **A+B+D** | 0.8511239 | **0.8446741** | 0.6622277 | 0.6996494 | 0.7622247 | 0.5652757 |
| A+B+G | 0.6769592 | 0.6851529 | 0.5039422 | 0.6995586 | 0.7128057 | 0.532324 |
| A+B | 0.8410205 | 0.8420532 | 0.6559716 | 0.6993441 | 0.7584764 | 0.565624 |
| D+F | 0.7890745 | 0.7828738 | 0.5883612 | 0.6970378 | 0.7481484 | 0.5527718 |
| A+D+G | 0.6850894 | 0.6962369 | 0.5098307 | 0.6962278 | 0.6984858 | 0.5170034 |
| E+F | 0.384148 | 0.4019777 | 0.2807841 | 0.6958143 | 0.6291915 | 0.4596991 |
| B+C+D+F+G | 0.7465695 | 0.7428089 | 0.5548506 | 0.6919732 | 0.7404214 | 0.5500107 |
| E | 0.3576092 | 0.4240361 | 0.2959173 | 0.6918483 | 0.654034 | 0.4840656 |
| A+E+F+G | 0.4808226 | 0.5494683 | 0.3864856 | 0.6917831 | 0.6596317 | 0.4885645 |
| A+B+C+D+F | 0.8447283 | 0.8382587 | 0.655438 | 0.6915678 | 0.74804 | 0.5520339 |
| A+C+D+F | 0.8296473 | 0.8267216 | 0.6432083 | 0.68574 | 0.7237892 | 0.5300617 |
| D+E+F+G | 0.5251757 | 0.5788689 | 0.4057678 | 0.6849524 | 0.6895598 | 0.5170181 |
| A+B+C+F | 0.8316004 | 0.8362747 | 0.6513356 | 0.6847957 | 0.7399676 | 0.5479594 |
| B+D+F+G | 0.6775829 | 0.6812851 | 0.4941444 | 0.6843386 | 0.7336601 | 0.5467848 |
| B+D+F | 0.8071171 | 0.8094019 | 0.6148455 | 0.6825941 | 0.7663726 | 0.5723973 |
| A+C+F+G | 0.6575887 | 0.6762202 | 0.4958229 | 0.6813433 | 0.6591407 | 0.4846894 |
| B+E+F+G | 0.4982775 | 0.5527718 | 0.3903376 | 0.6806216 | 0.7005131 | 0.5304855 |
| **A+B+C+D** | **0.8540436** | 0.8431603 | **0.6626772** | 0.6772973 | 0.7417459 | 0.5453327 |
| A+E+G | 0.472955 | 0.5530346 | 0.3890103 | 0.6771249 | 0.6573487 | 0.4893027 |
| B+C+D+G | 0.7505271 | 0.7464292 | 0.5598595 | 0.6755846 | 0.7358615 | 0.5443092 |
| B+C+F+G | 0.6759061 | 0.6788792 | 0.4979017 | 0.6720992 | 0.7086397 | 0.5229909 |
| C+D+F+G | 0.6836245 | 0.6946723 | 0.5098541 | 0.6712161 | 0.6964381 | 0.510346 |
| A+C+D | 0.8439398 | 0.8332586 | 0.6511831 | 0.6691492 | 0.7161186 | 0.5225399 |
| A+B+C | 0.8485692 | 0.8430315 | 0.6604375 | 0.6676815 | 0.7321051 | 0.5404549 |
| B+F | 0.7516723 | 0.7666937 | 0.5673202 | 0.6667692 | 0.7619533 | 0.5776199 |
| A+F | 0.6932569 | 0.7225984 | 0.5330767 | 0.6666441 | 0.6347037 | 0.4562851 |
| A+C+G | 0.6656358 | 0.6824908 | 0.4999738 | 0.6656107 | 0.6546479 | 0.4832854 |
| D+E+G | 0.516871 | 0.5697754 | 0.4000935 | 0.6651448 | 0.6804707 | 0.5099788 |
| B+C+D+F | 0.8247287 | 0.8184928 | 0.6297652 | 0.6626351 | 0.7400761 | 0.5445067 |
| B+D+G | 0.6753924 | 0.6808575 | 0.4944656 | 0.6608944 | 0.7249865 | 0.5380795 |
| B+E+G | 0.4877159 | 0.5410212 | 0.3818796 | 0.6594013 | 0.6905136 | 0.5246408 |
| C+E+F+G | 0.4891721 | 0.5728004 | 0.4008756 | 0.6591003 | 0.6635246 | 0.4917234 |
| D | 0.8126264 | 0.8070632 | 0.6214802 | 0.65596 | 0.7373704 | 0.5414952 |
| C+D+F | 0.8097343 | 0.8037049 | 0.6138982 | 0.6541172 | 0.7130854 | 0.5175723 |
| B+D | 0.8137247 | 0.8233155 | 0.6370843 | 0.6518691 | 0.7559907 | 0.5599014 |
| A+C+F | 0.7752843 | 0.7951124 | 0.6057329 | 0.6502271 | 0.6633348 | 0.4833917 |
| C+D+G | 0.6860892 | 0.6978751 | 0.5131768 | 0.6497435 | 0.6910838 | 0.5052927 |
| B+C+G | 0.6792946 | 0.6786792 | 0.4994426 | 0.6497126 | 0.7004212 | 0.5137811 |
| B+C+F | 0.8038699 | 0.8062633 | 0.6191791 | 0.6440506 | 0.7259662 | 0.5365903 |
| B+C+D | 0.8321197 | 0.8243253 | 0.6393731 | 0.640315 | 0.7314719 | 0.53465 |
| C+E+G | 0.4810865 | 0.5733334 | 0.4038344 | 0.6400271 | 0.6587451 | 0.4902983 |
| A | 0.7330521 | 0.7388047 | 0.5494716 | 0.6396967 | 0.6086288 | 0.4392761 |
| D+F+G | 0.5511325 | 0.5898712 | 0.4169908 | 0.6343252 | 0.650566 | 0.4791092 |
| A+F+G | 0.4875017 | 0.5587394 | 0.3955062 | 0.6276952 | 0.599814 | 0.4332668 |
| A+C | 0.8010281 | 0.8067553 | 0.619178 | 0.6273149 | 0.6503579 | 0.474706 |
| C+D | 0.824337 | 0.8135269 | 0.6273393 | 0.6250023 | 0.7056866 | 0.5098694 |
| B+F+G | 0.5144075 | 0.5630783 | 0.3975143 | 0.618648 | 0.6681596 | 0.503393 |
| B | 0.7718778 | 0.7966052 | 0.605009 | 0.6125134 | 0.7439667 | 0.5566676 |
| B+C | 0.8209444 | 0.813971 | 0.6317665 | 0.6114811 | 0.7137722 | 0.523751 |
| D+G | 0.5371244 | 0.577217 | 0.4081581 | 0.5973786 | 0.6300433 | 0.4616126 |
| A+G | 0.4730156 | 0.5527451 | 0.3903595 | 0.5944861 | 0.5823288 | 0.4204908 |
| C+F+G | 0.5032671 | 0.5833657 | 0.4123181 | 0.5853725 | 0.6068769 | 0.4383447 |
| C+F | 0.7142169 | 0.7405708 | 0.5496908 | 0.5774712 | 0.6153675 | 0.4441273 |
| B+G | 0.4954585 | 0.5422993 | 0.3785361 | 0.5760827 | 0.6433016 | 0.482002 |
| E+F+G | 0.2478509 | 0.3174246 | 0.2195575 | 0.5492813 | 0.5313104 | 0.3853897 |
| C+G | 0.4885831 | 0.576078 | 0.4107611 | 0.543921 | 0.5910136 | 0.4296071 |
| C | 0.7527691 | 0.7517052 | 0.5701154 | 0.5195201 | 0.5853968 | 0.423547 |
| E+G | 0.2086825 | 0.3326879 | 0.2251188 | 0.5052977 | 0.5428208 | 0.3942009 |
| F | 0.3039538 | 0.2872049 | 0.1949141 | 0.4578741 | 0.4392104 | 0.3050477 |
| F+G | 0.155876 | 0.2554586 | 0.1760319 | 0.4000613 | 0.4607525 | 0.3219535 |
| G | 0.08441809 | 0.2420689 | 0.158013 | 0.3073668 | 0.42897 | 0.3069002 |

**Supplementary Table 4.** Cumulative observed global interface and fold scores of the top raked models for every combination of the 7 component scores based on the CASP15 multimer data: A=ModFOLDIA, B=DockQJury, C=QSscoreJury, D=QSscoreOfficialJury, E=lDDTOfficialJury, F=voronota-js-voromqa, G=CDA-score. The table is sorted by the cumulative global fold score. The top scores in each column are shown in bold. The ModFOLDdockR fold and interface score combinations are highlighted in green.

| Method combination | Interface | Fold |
| --- | --- | --- |
| **A+B+D+E+F+G** | **22.135** | **30.0735** |
| A+B+D+E+F | 22.045 | 30.047 |
| A+B+D+F+G | 21.816 | 29.399 |
| B+D+E | 21.129 | 29.3515 |
| A+C+E+F+G | 21.2075 | 29.3475 |
| A+D+E+F+G | 21.419 | 29.345 |
| B+E | 21.0365 | 29.3345 |
| A+C+E+F | 21.222 | 29.293 |
| B+E+F | 21.366 | 29.287 |
| A+E | 20.805 | 29.269 |
| B+E+G | 21.173 | 29.2425 |
| B+D+E+F | 20.955 | 29.2415 |
| B+D+E+G | 21.197 | 29.188 |
| A+B+D+F | 21.762 | 29.172 |
| A+D+E+F | 21.357 | 29.1695 |
| A+E+G | 20.8805 | 29.1685 |
| B+E+F+G | 21.3325 | 29.139 |
| A+B+C+F+G | 21.331 | 29.137 |
| A+E+F | 20.792 | 29.119 |
| B+D+E+F+G | 21.163 | 29.112 |
| A+E+F+G | 20.7765 | 29.111 |
| A+B+E+F+G | 21.1915 | 29.023 |
| A+B+C+F | 21.21 | 29.0215 |
| B+D+F+G | 21.089 | 29.0065 |
| A+D+F | 20.7175 | 28.9995 |
| A+B+E+F | 21.109 | 28.9895 |
| D+E | 20.8495 | 28.982 |
| A+D+E | 21.244 | 28.945 |
| A+B+D+E | 21.2275 | 28.9405 |
| D+E+G | 20.878 | 28.917 |
| B+D+F | 21.254 | 28.867 |
| A+B+D+E+G | 21.185 | 28.8365 |
| A+D+E+G | 21.2255 | 28.7915 |
| D+E+F | 20.45 | 28.7735 |
| A+D+F+G | 20.7675 | 28.7725 |
| E | 20.5455 | 28.7715 |
| E+F | 20.029 | 28.707 |
| D | 20.7635 | 28.62 |
| A+B+E | 21.023 | 28.6195 |
| A+B+F+G | 20.6245 | 28.597 |
| A+B+C+G | 21.273 | 28.5665 |
| A+B+C+E | 20.2525 | 28.566 |
| B+C+E+F+G | 20.1145 | 28.5555 |
| B+D+G | 20.8825 | 28.5555 |
| A+C+D+E+F | 20.453 | 28.5455 |
| A+B+E+G | 20.9875 | 28.5445 |
| A+B+C+E+F+G | 20.4425 | 28.5235 |
| A+B+F | 20.971 | 28.5205 |
| D+G | 20.984 | 28.497 |
| B+C+D+E+F | 20.191 | 28.4865 |
| A+B+C+D+E+F | 20.6115 | 28.486 |
| A+B+C+E+F | 20.372 | 28.4715 |
| A+C+D+G | 20.909 | 28.4705 |
| A+B+D+G | 20.8465 | 28.4575 |
| A+B+C+D+F | 21.058 | 28.4555 |
| A+C+D | 20.808 | 28.446 |
| E+G | 19.9055 | 28.4435 |
| B+C+E+F | 20.0165 | 28.435 |
| A+B+C | 21.074 | 28.4115 |
| D+F | 20.3295 | 28.3715 |
| D+E+F+G | 20.235 | 28.371 |
| A+D | 20.558 | 28.358 |
| C+D+E+F | 20.1225 | 28.345 |
| B+F | 21.298 | 28.3385 |
| A+D+G | 20.6425 | 28.33 |
| B+C+E | 19.98 | 28.322 |
| A+C+D+F | 20.72 | 28.314 |
| A+C+F | 20.3935 | 28.312 |
| A+C+D+F+G | 20.7665 | 28.297 |
| A+B+C+D+G | 20.9835 | 28.287 |
| C+D+E+F+G | 20.08 | 28.2725 |
| B+D | 20.97 | 28.271 |
| A+B+C+D+F+G | 20.8845 | 28.2405 |
| C+E+F | 19.1805 | 28.236 |
| A+B+C+D+E+G | 20.5715 | 28.2305 |
| B+F+G | 20.979 | 28.2285 |
| A+C+E | 20.261 | 28.2185 |
| A+B+D | 20.8275 | 28.214 |
| E+F+G | 20.0295 | 28.211 |
| B | 21.285 | 28.2015 |
| A+B+C+D+E | 20.467 | 28.1905 |
| A+B+C+E+G | 20.3195 | 28.1855 |
| D+F+G | 20.4925 | 28.185 |
| B+C+D+F | 20.0495 | 28.1805 |
| A+C+D+E+F+G | 20.3585 | 28.165 |
| A+F | 19.8055 | 28.126 |
| A+C+E+G | 20.3095 | 28.11 |
| A+B+C+D+E+F+G | 20.473 | 28.0795 |
| B+C+D+E+F+G | 20.1685 | 28.0715 |
| A+C+D+E | 20.3475 | 28.0645 |
| A+B+C+D | 20.972 | 28.0445 |
| C+D+E+G | 19.967 | 28.044 |
| B+C+D+E | 19.9605 | 28.025 |
| A+C+F+G | 20.5735 | 28.021 |
| C+D+E | 19.9275 | 28.013 |
| A+B+G | 20.5985 | 28.009 |
| B+C+D+E+G | 20.0565 | 28.0085 |
| B+C+D+F+G | 20.0345 | 28.008 |
| A+C+D+E+G | 20.349 | 27.9955 |
| A+B | 20.581 | 27.934 |
| B+C+E+G | 19.862 | 27.8685 |
| C+D | 19.3885 | 27.861 |
| A+F+G | 19.717 | 27.857 |
| F | 21.1 | 27.8555 |
| C+D+G | 19.501 | 27.8505 |
| B+C+D+G | 20.146 | 27.8225 |
| C+D+F | 19.8945 | 27.802 |
| A+C+G | 20.537 | 27.771 |
| A | 19.4745 | 27.7495 |
| C+D+F+G | 19.791 | 27.7435 |
| B+C+D | 20.0025 | 27.6155 |
| C+E+F+G | 18.3595 | 27.59 |
| A+C | 20.3035 | 27.5675 |
| C+E+G | 18.4495 | 27.551 |
| C+E | 18.405 | 27.394 |
| B+C+F | 19.275 | 27.193 |
| A+G | 18.7035 | 27.065 |
| B+C+F+G | 19.121 | 26.9555 |
| B+G | 19.6465 | 26.839 |
| B+C | 18.7555 | 26.532 |
| B+C+G | 18.681 | 26.3425 |
| F+G | 17.531 | 25.616 |
| C+F | 15.2185 | 24.9425 |
| C+F+G | 15.3935 | 24.835 |
| G | 12.9265 | 23.4585 |
| C | 13.147 | 23.291 |
| C+G | 13.1915 | 23.073 |
